# Attractor Concepts to Evaluate the Transcriptome-wide Dynamics Guiding Anaerobic to Aerobic State Transition in *Escherichia coli*

**DOI:** 10.1038/s41598-020-62804-3

**Published:** 2020-04-03

**Authors:** Thuy Tien Bui, Kumar Selvarajoo

**Affiliations:** 1 Singapore Institute of Food & Biotechnology Innovation, Agency for Science, Technology and Research A*STAR, Proteos, 61 Biopolis Drive, 138673 Singapore, Singapore; 2 Synthetic Biology for Clinical and Technological Innovation (SynCTI), National University of Singapore, 28 Medical Drive, 117456 Singapore, Singapore

**Keywords:** Computational biology and bioinformatics, Systems biology

## Abstract

For any dynamical system, like living organisms, an attractor state is a set of variables or mechanisms that converge towards a stable system behavior despite a wide variety of initial conditions. Here, using multi-dimensional statistics, we investigate the global gene expression attractor mechanisms shaping anaerobic to aerobic state transition (AAT) of *Escherichia coli* in a bioreactor at early times. Out of 3,389 RNA-Seq expression changes over time, we identified 100 sharply changing genes that are key for guiding 1700 genes into the AAT attractor basin. Collectively, these genes were named as *attractor* genes constituting of 6 dynamic clusters. Apart from the expected anaerobic (glycolysis), aerobic (TCA cycle) and fermentation (succinate pathways) processes, sulphur metabolism, ribosome assembly and amino acid transport mechanisms together with 332 uncharacterised genes are also key for AAT. Overall, our work highlights the importance of multi-dimensional statistical analyses for revealing novel processes shaping AAT.

## Introduction

Microorganisms are able to adapt to diverse environmental changes, making them the longest surviving living systems on this planet. The well-studied bacterium *Escherichia coli* is able to switch between 2 stable attractor states, aerobic and anaerobic conditions, based on oxygen availability^1^. *E. coli* grows well on both conditions, albeit at a lower rate anaerobically. Over the last few decades, numerous studies have investigated the transition of *E. coli* between the states from a traditional and reductionist standpoint^2,3^.

The current understanding of *E. coli* metabolism during aerobic condition is that the glucose flux moves through the glycolysis pathway, and channelled to the TCA cycle via pyruvate dehydrogenase complex. This mode of respiration yields higher ATP levels, thereby, generating more energy compared to anaerobic respiration. In the absence of oxygen, depending on the availabilities of electron donors or acceptors, pyruvate formate-lyase, nowadays called formate C-acetyltransferase, catalyses pyruvate and coenzyme-A into formate and acetyl-CoA, a reversible conversion. In addition, lactate dehydrogenase also acts on pyruvate to produce lactate, and still others include succinate, acetate and ethanol production. These processes, collectively, suppress the metabolic fluxes channelling to the TCA cycle. Thus, major metabolic switching mechanisms occur during aerobiosis or AAT, and numerous recent studies have focused on deciphering other key novel mechanisms that are also concerted. For example, Green and colleagues used microarray transcript profiling to reveal peroxide stress response and methionine biosynthesis as novel processes induced during aerobiosis^4^. Although these works have shed light into the novel processes guiding *E. coli* AAT, it is important to note that living cells are dynamical systems that involve large interplay of cellular networks.

For understanding complex cellular behaviours such as immune response or growth, numerous studies have employed computational models utilizing linear and non-linear differential equations to monitor intracellular as well as extracellular molecular species, such as proteins or metabolites turnover, over time^5^. In addition, dynamical systems and chaos theories have also been used to study the complex self-organizing (e.g. skin pattern formation) and stochastic or chaotic behaviors (e.g. multiple lineage cell differentiation from single cell origin) of living systems^6^. Here, the models developed are qualitative, rather than quantitative, in defining the dynamical system, and are often used to understand the sensitivity of the initial conditions, or perturbations, to their longer term steady or periodic states^7^.

**Figure 1:**
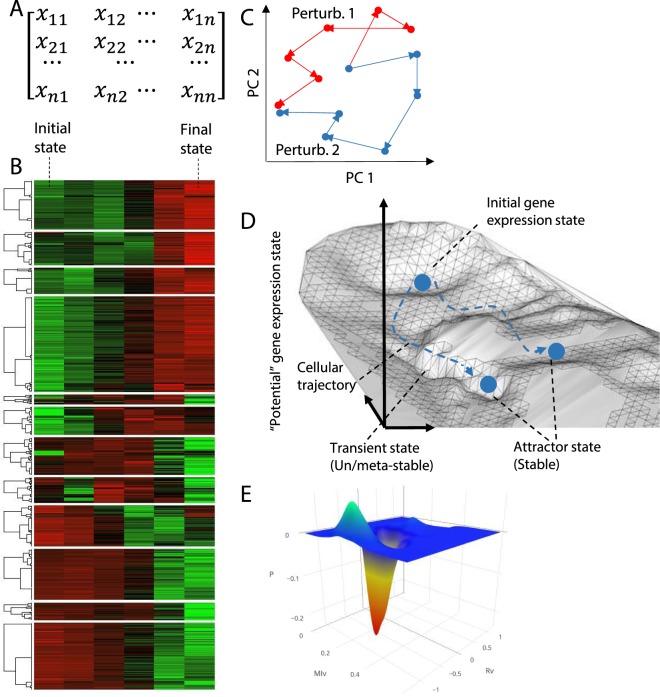
Schematic illustration of attractor landscape and cellular trajectory. (**A,B)** Cellular gene expression profile represented by **(A)** matrix and **(B)** heat map of gene expression level. Each element represents the expression level of a gene (rows) in a cellular state (columns). **(C)** Schematic representation of cell trajectory convergence on principal components 1 and 2 (denoted as PC1 and PC2) space. Each point represents a sample’s entire gene expression profile within one of the two transitioning processes caused by two distinct perturbations^13^. **(D)** The landscape is a schematic 3-D projection of *N* (total number of genes) to a two-dimensional state space. In the attractor landscape, many stationary attractors (represented by the local minima), which correspond to the natural cellular phenotypes such as cell fate, might co-exist. Each attractor associates with a unique cellular signature profile (or gene expression profile in this study). The transitioning processes (dashed blue line) guide the cell from one stable attractor to another^57^. **(E)** Gene expression attractor landscapes generated by the superimposition of probability distribution of Pearson and mutual information correlation mertics to create a 3-D space^18^. Existence of stable attractor coinciding the convergence of cellular trajectories is indicated by the local minima.

With the recent advent of high-throughput technologies, we are now able to observe transcriptome-wide gene expression changes over time as opposed to only a few proteins or metabolites monitored traditionally. We also know that not individual genes, but rather the complex networks of genes drive key cellular processes. Therefore, genes can be considered to be a set of points in *state space*^8,9^. The *state* refers to the expression vector of a large set of co-regulated genes of interest. In other words, the alteration of expression levels of specific sets of genes can be represented by a continuous trajectory in their expression state space^10^. Some of these trajectories are *attractive*, that is, they converge towards a fixed point if the system is perturbed from a nearby state (Fig. 1).

Each cellular phenotype can correspond to an *attractor* in the gene expression state space, where they represent stable state, either peaks or valleys in the landscape, to which the system returns given a small biological perturbation^11^. In other words, the dynamical pattern of gene expressions developing over time reflect the concerted regulation of the transcriptome and can lead to convergence or *equilibrium* of the cell state, often referred to as *attractor* state^12,13^.

The presence of multi-dimensional *attractor* states based on high-throughput gene expression data has been experimentally implicated in recent times^14,15^. It has been shown that dynamic gene expression trajectories, using principal components and correlation metrics, reveals subset of the state space convergence in time, a hallmark of attractor, coinciding with the end of cell fate completion^16–18^ (Fig. 1C,D).

Despite the progresses made for *E. coli* AAT, as mentioned earlier, detailed gene expression response using multi-dimensional statistical approaches and a global transcriptome-wide scale analyses using dynamical system approach still remain largely underexplored. Most studies investigate only the most differentially expressed genes over time using arbitrary expression fold, e.g. 2-fold, cut-off without investigating the underlying statistical structure or undertake dynamical systems view. However, during aerobiosis or cell state changes, the transcriptomic network invokes a progressive directional change of thousands of gene expressions in time, through which the cell state expression pattern is adopted^19–21^.

With the recent advances in understanding how transcriptomic networks govern cell fate decisions, it is now possible to explore biological patterns and *order* using multi-dimensional statistics^22–25^. Here we, therefore, extended the concept, using multi-dimensional statistical methods on RNA-Seq data, to investigate the anaerobic to aerobic transition of *E. coli* grown in 3-liter bioreactors. Sampling of cells for transcriptome analysis were obtained rapidly, with 6 time points in the first 10 minutes. Using distribution fitting, linear and non-linear correlations (to develop attractor landscape), PCA (to observe attractor trajectory), hierarchical clustering and gene ontology, we report the gene expression patterns and functions during early aerobiosis.

## Results

### *E. coli* transcriptome-wide expressions follow lognormal distribution

The original experiments were performed by Feuer and colleagues^26^. Briefly, *E. coli* K-12 strain W3110 cells were grown anaerobically in a 3-liter continuously stirred tank bioreactor at pH7 and 37°C, and stirred at 500 rpm with a Rushton turbine. The first sample was drawn (*t* = 0) when OD of 3 at 600 nm was achieved, and air supply of 1L/min was then initiated. Subsequent samples were taken at *t* = 0.5, 1, 2, 5 and 10 min, with 3 biological replicates. The resultant RNA-Seq data, in read counts, for all samples were deposited and available with GEO accession number GSE71562.

**Figure 2:**
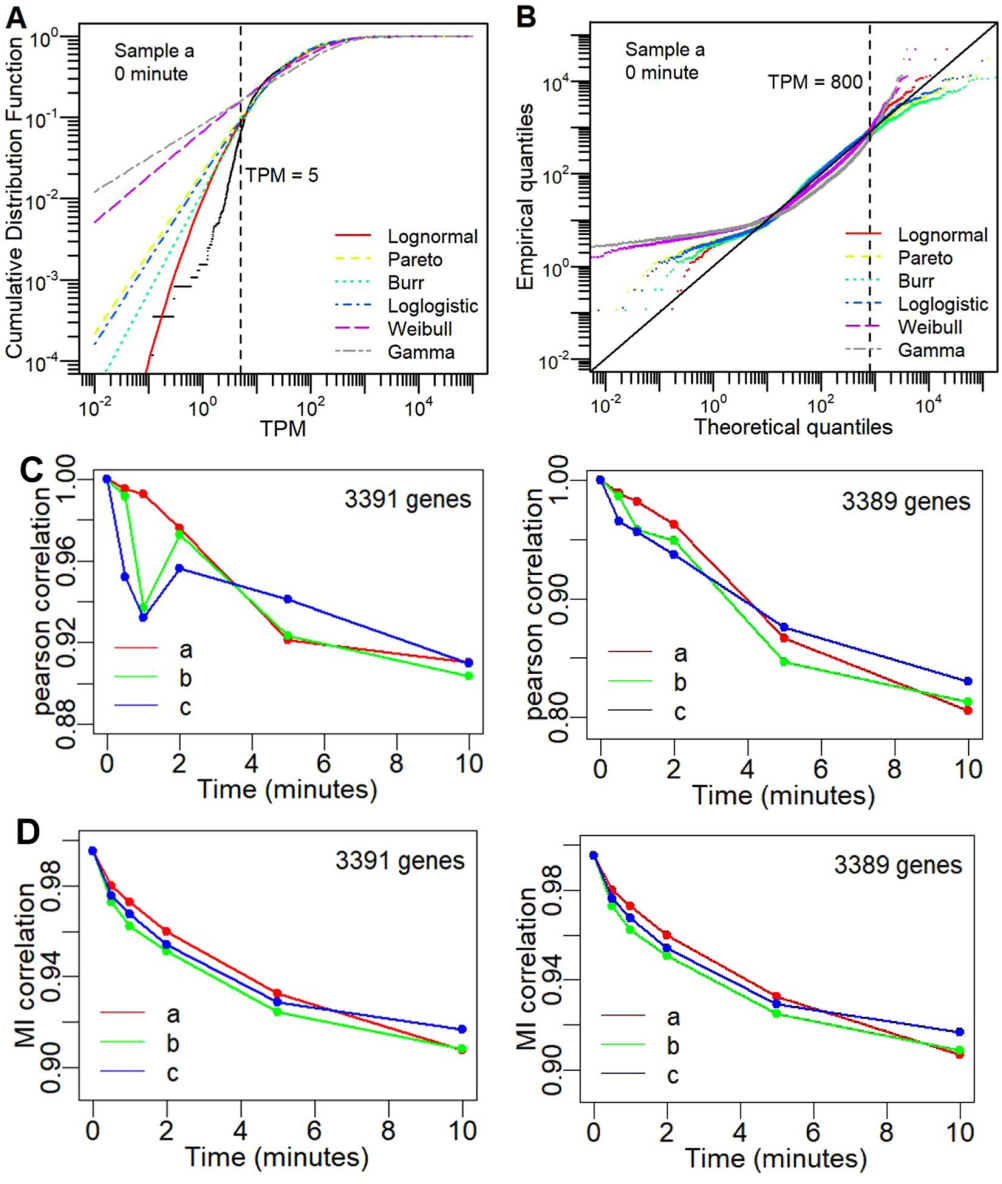
*E. coli* transcriptome-wide statistical properties. (**A**,**B**) Comparison of transcriptome-wide data with statistical distributions: **(A)** Cumulative distribution functions versus TPM values in logscale, and **(B)** Quantile-quantile plot between transcriptome data (experimental data – black colour) and lognormal (red), Pareto (yellow), Burr (cyan), loglogistic (blue), Weibull (purple), and gamma (grey) statistical distributions (Methods). Figure is one representative at *t* = 10 min for replicate *a*. See Fig. S1 for other time points and replicates. Transcriptome-wide correlation in time using: **(C)** Pearson correlation, and **(D)** Mutual Information-based correlation metrics (Methods) between time *t*_0_ (0 min) and *t*_*i*_ (0, 0.5, 1, 2, 5, 10 min) for all 3 replicates (replicate a – red, replicate b – green, replicate c - blue) across 3391 genes with expression above 5 TPM (left panels) and 3389 genes upon removal of two highest expressed genes *rnpB* and *lpp* (right panels).

The RNA-Seq data needed to be checked and reduced for gene expressions that can be considered noisy, especially the lowly expressed ones. Previously, we had used statistical distributions on normalized expressions as a means to remove unreliable or noisy genes^17,23,24,27^. Therefore, Transcripts Per Kilobase Million or Transcripts Per Million (TPM) normalization of the read counts of all samples were performed and checked for statistical distribution of all (4240 non-zero) transcripts for all time points (Figs. 2A and S1A). As previously seen for other cell types, we observed theoretical Pareto (power-law) and lognormal distributions best followed the experimental transcript distributions above a threshold of 5 TPM. Next, to check how close the gene expressions match the Pareto and lognormal distributions, we performed a Quantile-Quantile plot (Figs. 2B and S1B). The data show that lognormal has major advantage over Pareto, noticeable for the higher expressions (TPM > 800). The quality of statistical distribution fit was finally confirmed using the Akaike information criterion (Table S1). Notably, lognormal distribution for gene expressions has also been recently implicated for several other cell types^27,28^. Hence, we concur that our *E. coli* gene expressions follow lognormal distribution across all replicates and time points (Fig. S1A,B) and retained genes with transcript levels above the 5 TPM threshold for further analysis (*N* = 3391).

### Correlation analysis shows gradual transcriptome-wide deviation from origin

Temporal correlation metrics are often used to check how a dataset deviates from their initial condition, time or perturbation^17,18,22^. Here, to investigate the transcriptome-wide deviation from *t* = 0, we adopted 4 correlation metrics (Pearson, Spearman, Biweight midcorrelation or bicor^29^, and Mutual Information-based correlation^30^ *MI*_*c*_, see Methods). We used these metrics to investigate parametric (Pearson), and non-parametric (Spearman, bicor and *MI*_*c*_) correlations. All metrics pointed to a gradual decay in the transcriptome-wide correlation with time for all 3 replicates (Fig. 2C,D – left panel and Fig. S2). These data suggest that there is a constant and gradual global transcriptomic “movement” in time^31^.

Looking closer, the Pearson correlation, which measures the linear association between 2 vectors, showed a sharp initial decay (*t* = 0.5 and 1 min) and a recovery before gradual decay for 2 replicates. This “discrepancy” is absent in the other 3 non-parametric correlations which could be less outlier-sensitive. In addition, the Q-Q plots show that the highly expressed genes deviated from the global statistical distribution (Figs. 2B and S1B). Thus, we removed genes one by one from the highest expression and checked the transcriptome-wide Pearson correlation. Consequently, the removal of the top 2 highest expressed genes (*rnpB* and *lpp*) was sufficient to result in smooth correlation decay in all metrics used (Fig. 2C,D – right panel and Fig. S2). Thus, we kept the remaining genes (*N* = 3389) for further analysis. Next, we investigate which portions of genes are key for guiding the anaerobic to aerobic state transition. To do this, we need to consider the concepts of attractors often used in physics and mathematics^18,32^.

### Defining attractor region and transcriptomic elements

State space for a dynamical system is the set of all possible states, where each state of the system corresponds to a unique point in the state space^13,14,20^. As it is difficult to define explicit expressions for representing transcriptome-wide dynamics, the analysis of state space as set of all possible pairs of linear and non-linear gene expression correlation dynamics (based on *R*_*v*_ and *MI*_*v*_, see below) provides a useful way for understanding the qualitative features of attractor localizations or visualizations^18,22^. Note that the modified mutual information (*MI*_*v*_) and Pearson correlation (*R*_*v*_) used here are slightly different from those used in the previous section (Method).

**Figure 3:**
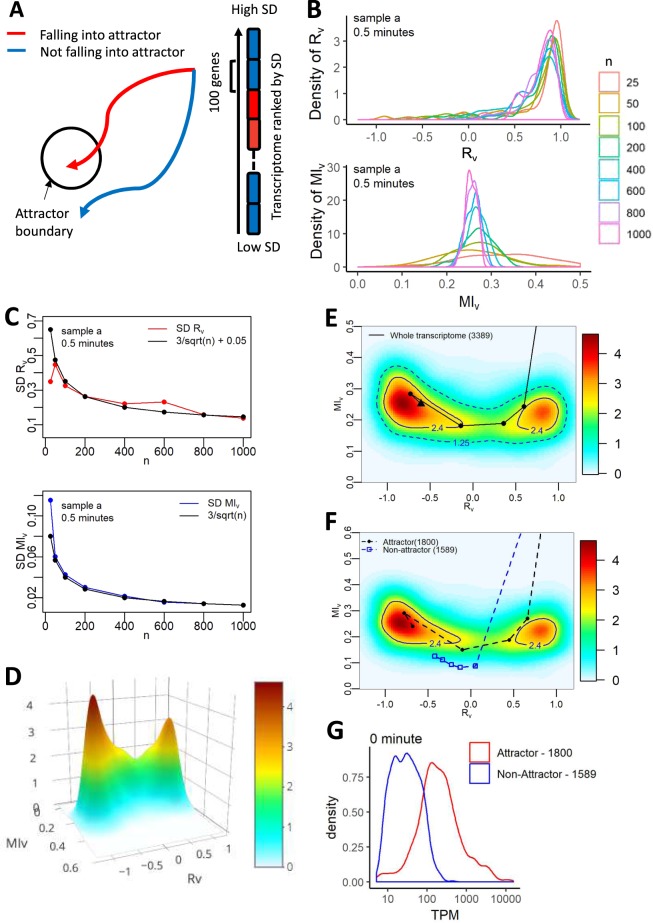
Attractor landscape by probability density distributions of correlations, transcriptomic elements and *attractor* genes. (**A)** Schematic trajectories for transcriptomic elements falling into attractor (red) and not falling into attractor (blue). **(B)** Distribution and **(C)** Standard deviation of *R*_*v*_ (top panel) and *MI*_v_ (bottom panel) with different transcriptomic element size (denoted as *n*) of replicate *a* at 0.5 minutes. Distribution of *R*_*v*_ and *MI*_*v*_ for ensembles of *n* randomly chosen genes (*n* = 25, 50, 100, 200, 400, 600, 800, 1000) were generated with 100 repeats. Standard deviation of *R*_*v*_ and *MI*_*v*_ decreases as *n* increases (except for when *n* = 25 for *R*_*v*_), and follows $\alpha /\sqrt{n}$ + c law. See Fig. S3A,B for other time points. (**D**) 3D plot of the superimposition of the probability distribution (SPD) of *R*_*v*_ and *MI*_*v*_ over all time points for the whole transcriptome. SPDs were estimated by getting *R*_*v*_ and *MI*_*v*_ values of 100 randomly chosen genes for 100 times, using two-dimensional kernel density estimation. **(E)** Trajectory of *whole genome* (3389 genes) falling within attractor boundary (solid contour line) overlaid on SPD of whole transcriptome *R*_*v*_ and *MI*_*v*_. The trajectory was generated by averaging 100 trajectories of 100 randomly chosen genes from the pool of 3389 genes. **(F)** Trajectories of cumulative *attractor* (1800), and *non-attractor* (1589) genes overlaid on SPD of *R*_*v*_ and *MI*_*v*_ for whole transcriptome. **(G)** Distribution of expression level for *attractor* (red), and *non-attractor* (blue) genes at representative *t* = 0. See Fig. S3D for other time points.

The fractal nature of transcriptomic response in cell fate determination has been explored in previous studies^18,33,34^. Basically, discrete subsets of transcriptome are responsible for guiding the transition of gene expressions from one “equilibrium” attractor state to another. The attractor state is the result of the convergence of gene expression dynamics across the transitioning time. Thus, in order to identify the genetic drivers of AAT, we divided the transcriptome into discrete fractions, namely transcriptomic elements^18^, and compared their individual trajectories against attractor region (Fig. 3A). Any element falling within the attractor boundary is attributed to AAT response.

A transcriptomic element is a minimum set of genes that possess enough correlation information (based on *R*_*v*_ and *MI*_*v*_) to show whether they fall into a cell attractor state^18^. To identify the size of a transcriptomic element, *n* genes were randomly chosen from the whole genome (*n* = 25, 50, 100, 200, 400, 600, 800, 1000), and their *R*_*v*_ and *MI*_*v*_ were evaluated for 100 repeats at all time points. The standard deviation of the *R*_*v*_ and *MI*_*v*_ were then plotted for the different *n* sizes and compared with the law of large numbers or LLN^18,35^. Both *R*_*v*_ and *MI*_*v*_ distributions of the transcriptomic elements converged to specific values for *n* > 50 (Figs. 3B and S3A). Furthermore, the result shows that the standard deviations of *R*_*v*_ and *MI*_*v*_ distributions at each time point decrease with increasing *n*, approximately following the law of large number (Figs. 3C and S3B). To investigate ensemble of genes that fall into the AAT attractor basin we, therefore, chose *n* = 100 as a compromise between density distribution and to retain a reasonable number of transcriptomic elements since higher *n* reduces the total number of elements.

To check the existence of cell attractor state in AAT, we analysed the superimposition of probability density (SPD) distribution for modified Pearson correlations (*R*_*v*_) and modified mutual information (*MI*_*v*_) of gene expression deviations from *t* = 0 to 10 minutes^18^ (Method). We found 2 distinct peaks (Fig. 3D,E), with the major peak coinciding with the state transition end time of the experiments. This implies the existence of cell attractor by the localizations of *R*_*v*_ and *MI*_*v*_ on the correlation space. As previously, we defined the attractor region boundary or basin as the inflection plane of the major peak curves^18^, and observed the transcriptome-wide trajectory also fall within this attractor region (Fig. 3E). Note that there are usually several meta-stable attractor states before the final state transition occurs. Here, we are evaluating the early meta-stable attractor states crucial for AAT.

### Identification of genes falling into attractor basin

To identify the number of genes that fall into the attractor basin and their names, we first ranked all genes according to their expression variability, measured by standard deviation (SD) across time^18^. Secondly, we assembled the ranked genes into transcriptomic elements (*n* = 100) and checked their *R*_*v*_-*MI*_*v*_ trajectories in time across the coordinates of the attractor region (Fig. S4A). We observe that one element, with the highest SD, fall above the attractor basin, 4 elements (3, 4, 5 and 12) fall into the basin and the rest fall out and remain below the basin (Fig. S4A).

The Euclidean distance of each element compared with the whole transcriptome trajectory is shown in Fig. S3C. Notably, the elements that fall above and into the basin shows the closest distance (less than the mean values) with whole transcriptome trajectory. Thus, we concur that these 5 transcriptomic elements are key in shaping the AAT response, and checked their combined effect. As anticipated, merging the elements into a larger sub-transcriptome resulted in them falling into the attractor region (Fig. S3C, black dotted). Hence, we named them as *attractor* genes.

Among the remaining 29 transcriptomic elements, 13 show close Euclidian distance to the whole transcriptome trajectory (Fig. S3D, empty circle symbol), while 16 elements have large deviation (Fig. S3D. empty square symbol). We considered them as *pseudo-attractor* and *non-attractor* elements as their collective or combined *R*_*v*_-*MI*_*v*_ trajectories fall into and outside the attractor basin, respectively (Fig. S3D, green and blue dotted).

Finally, we combined the *attractor* (500) and *pseudo-attractor* (1300) genes and tracked their overall trajectories which resulted in them falling into the attractor basin (Fig. 3F). Since the 1,800 genes collectively enter into the attractor basin, we now re-term them as the *attractor* genes, while keeping the remaining as the *non-attractor* genes (Fig. 3F). In other words, our attractor analyses reveal 53% or about half of the transcriptome, spreading across a wide spectrum of expression levels (Figs. 3G and S3D), is responsible for shaping the *E. coli* AAT. Next, we also conducted a similar attractor study, sorting elements according to fold-changes instead (Fig. S4B). Here, we obtained 65% of the transcriptome fall into the attractor basin.

Overall, contrary to the general impression that only a small number of highly expressed genes shape AAT, our data suggest that gene expression levels are not indicative of AAT, and an order of about half the transcriptome is crucial for the biological state transition.

### Principal component analysis as a test for attractor genes

**Figure 4:**
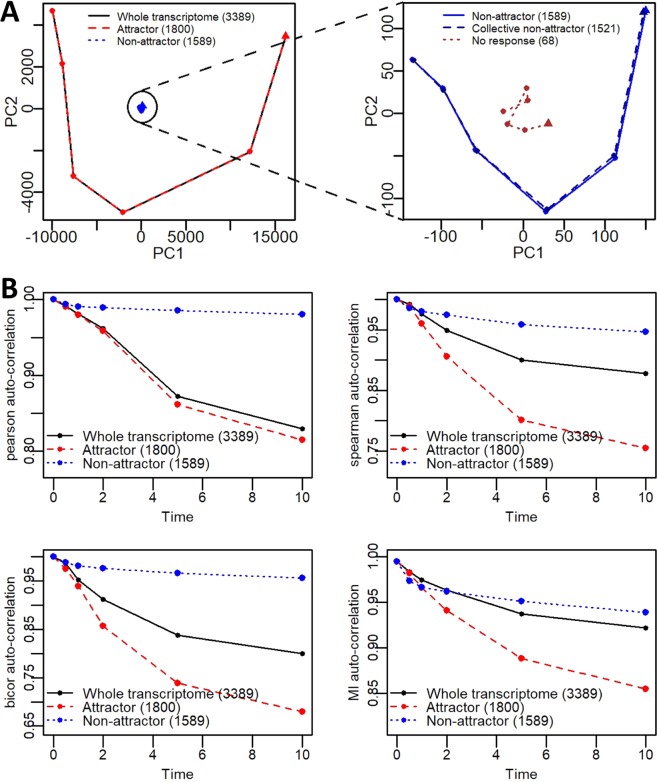
Principal component (PC) analysis and auto-correlations of whole transcriptome *attractor* and *non-attractor* genes. (**A)** Gene expression trajectory of *whole transcriptome* (black), *attractor* (red), *non-attractor* (blue), and *no response* genes (brown), obtained by taking the average trajectories of 3 replicates, presented on first 2 principal components space. Right panel indicates *non-attractor* trajectory on a larger scale. **(B)** Temporal correlation of *whole transcriptome* (black), *attractor* (red), and *non-attractor* (blue) genes using Pearson (top left panel), Spearman (top right panel), Biweight midcorrelation (bottom left panel) and Mutual Information-based (bottom right panel) correlation metrics.

Previous works had utilised principal components (PC) trajectories to investigate the dynamic global response of cell differentiation^13,16–18,36^. Here, we performed similar analysis for *whole*, *attractor* and *non-attractor* genes. PCs 1 and 2 constituted over 70% variance at *t* = 0 for all genes (Fig. S5A) and, hence, we tracked their values at each time point (Fig. 4A). As anticipated, our results show that the *attractor* genes tracked similar *whole genome* PC trajectories in time.

Next checking correlations, the *attractor* genes produce the most variable response (Fig. 4B). Nevertheless, the *non-attractor* genes also show monotonic or gradual variation in time, suggesting that even these genes can support AAT response, albeit with lower kinetics. Therefore, in this remaining 1,589 genes, we removed 68 genes that had below 1.12 fold changes at any time point, and checked their PCA and correlation plots (Figs. 4A and S5B). Our data show that removing the 68 genes did not noticeably affect the PCA or correlation response. Although the 1521 genes did not enter the attractor basin, they still show gradual response in time. Thus, these were named as *collective non-attractor* genes.

To summarize, our analysis indicate that 1,800 *attractor* genes shape the global AAT response, while 1521 genes follow weak but collective global response with remaining 68 genes not having any response. (Note that these 68 genes are additional to the 851 genes already removed during the low and high expression filtering using lognormal distribution fitting.)

### Temporal groups of attractor genes and characterization of major biological processes

**Figure 5:**
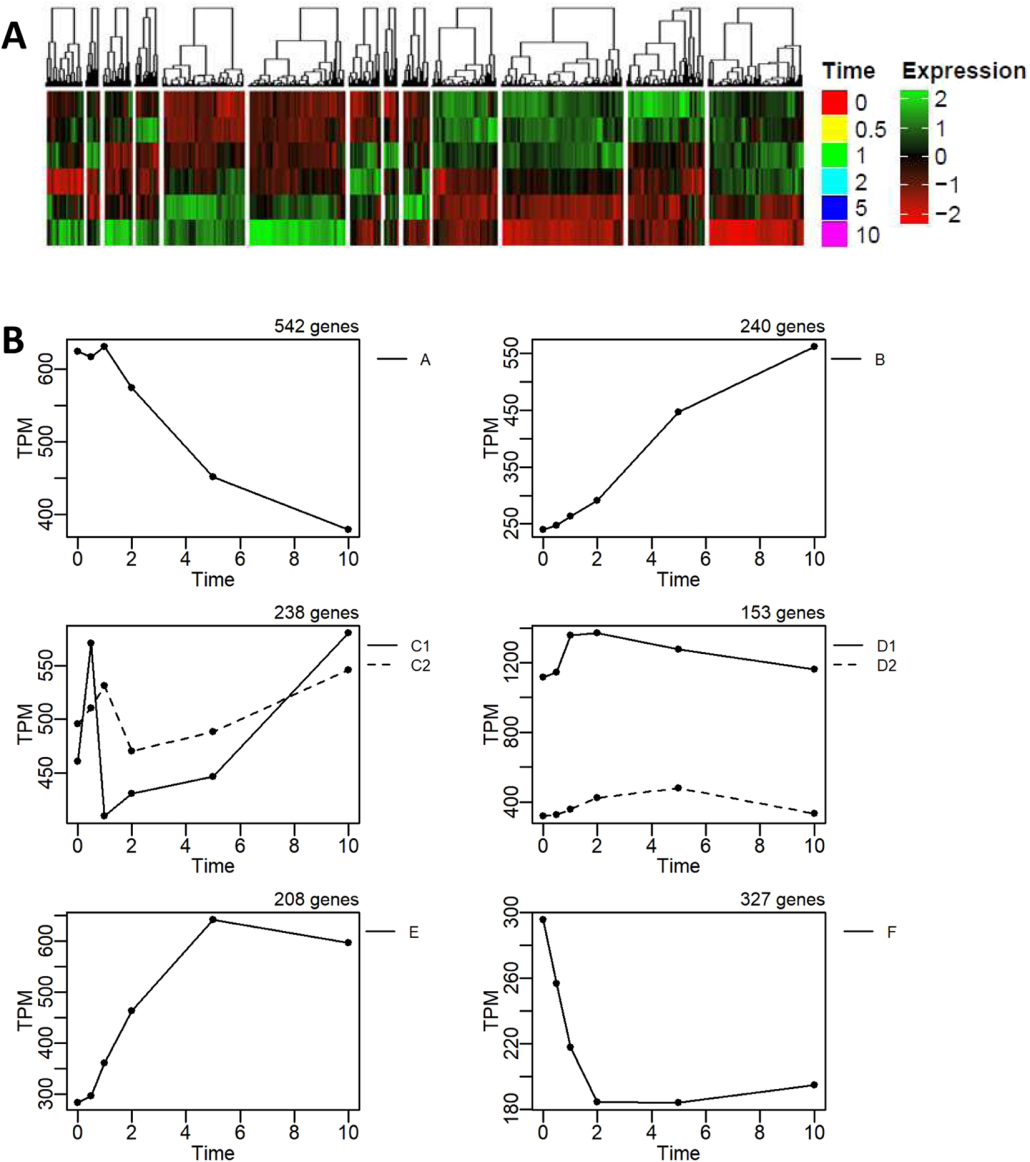
Major gene expression patterns of *attractor* genes. (**A)** Hierarchical clustering of *attractor* genes reveals 13 clusters of temporal expression profiles. **(B)** Six temporal average expression profile constructed by regrouping the 13 clusters: Group A: Gradual decay: Group B: Gradual activation; Group C: Fast activation, followed by decay and re-activation; Group D: Early activation, followed by decay; Group E: Early activation followed by plateau; Group F: Early decay, followed by plateau.

The previous section highlights the significance of the *attractor* genes in shaping the global response of *E. coli* in aerobiosis. To scrutinize the biological functions of *attractor* genes, hierarchical clustering was utilised, resulting in 13 initial clusters (Fig. 5A). From these clusters, we further refined the gene groupings by setting a Pearson correlation of above 0.7 between each gene’s temporal expression with the average profile for that group. Those that were below the threshold were re-evaluated within subgroups and subsequently re-grouped. As a result, we obtained 6 temporal groups of patterns for the *attractor* genes (Fig. 5B).

**Figure 6:**
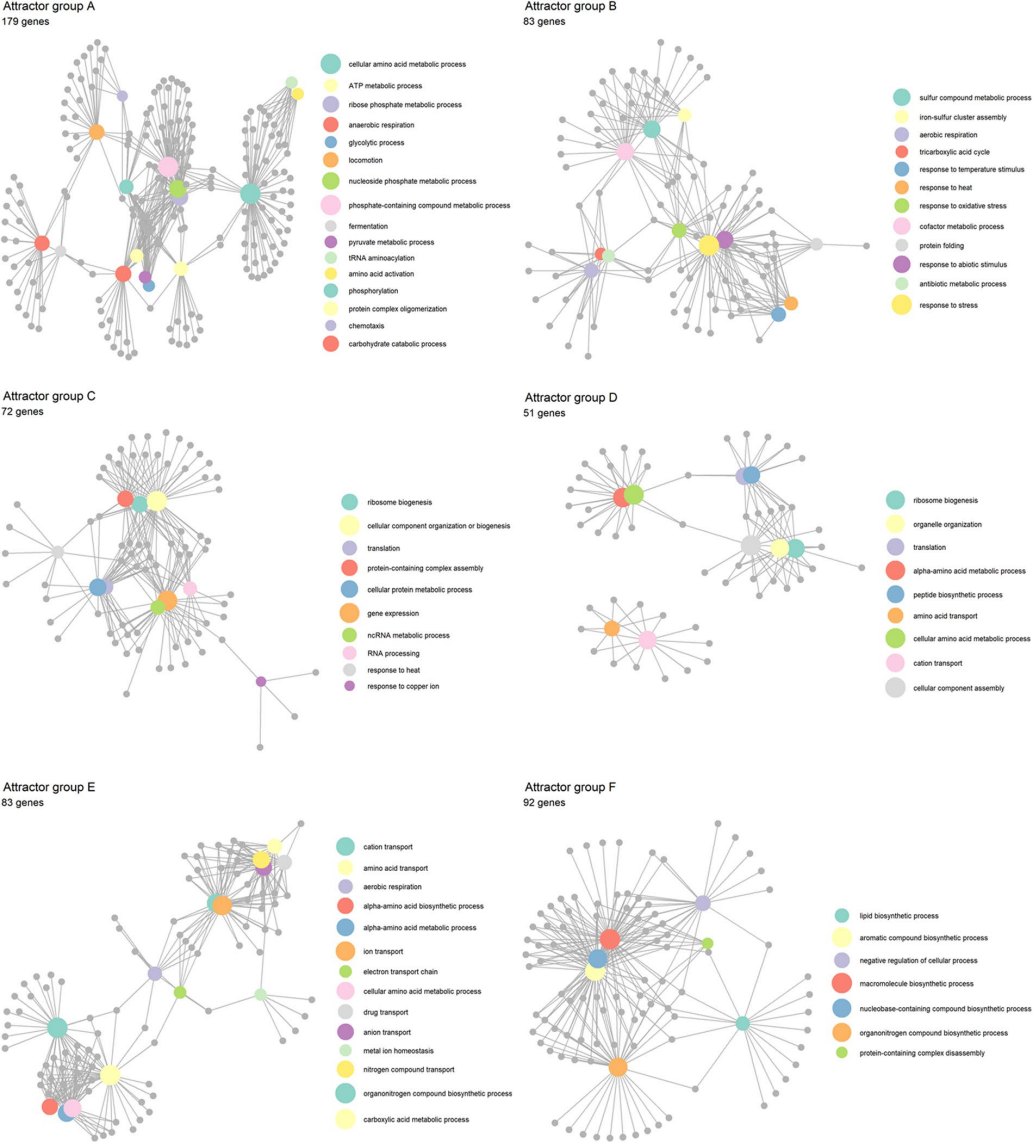
Selected enriched biological processes (coloured hubs) with their associated genes (grey dots) in the 6 major expression patterns of *attractor* genes. Full list of enriched processes is available in Table S2.

To elucidate the major biological processes that are regulated in each of the temporal groups, we performed Gene Ontology enrichment analysis using clusterProfiler R package^37^ with Entrez Gene database^38^ (both False Discovery Rate and adjusted *p*-value cut-off were set at 0.05). Enrichment analysis for the 6 groups of *attractor* genes resulted in (Fig. 6, Table S2): Group A: *Gradual decay*, mainly enriched in glycolysis, fermentation, anaerobic respiration, phosphorylation, amino acid metabolism, and locomotion, Group B: *Gradual activation*, enriched in TCA cycle, aerobic respiration, sulphur compound metabolism, protein folding and response to stress, Group C: *Fast activation, followed by decay and re-activation*, enriched in ribosome biogenesis, RNA processing, gene expression and response to copper ion, Group D: *Early activation*, *followed by decay*, mostly enriched in amino acid metabolism, cation transport, and organelle organization, group E: *Early activation followed by plateau*, enriched in aerobic respiration, several types of transporters, and ion homeostasis, and group F: *Early decay, followed by plateau*, enriched in biosynthesis of lipid, organonitrogen and nucleobase-containing compounds, and negative regulation of cellular processes.

Focusing on the names and functions of each *attractor* gene (using UniProt^39^ and EcoCyc^40^ databases), we observe several novel or uncharacterised genes for all groups (Tables S2–S4). Group A possesses several genes coding for flagellum protein, ligase, transcriptional regulator, hydrogenase/dehydrogenase functions, together with 71 uncharacterized genes. Group B contains ion and small molecular binding, and oxidoreductase genes together with 60 uncharacterized genes. Notably, a large portion of group C are tRNA genes (approximately 20% of the genes in group C) and ribosomal protein genes, with 76 uncharacterized genes. Group D consists of several ribosomal proteins (distinct from group C) and transporters, with 19 uncharacterized genes. Group E comprises mainly of oxidoreductase, ion transferase and binding proteins, with 33 uncharacterized genes. Finally, group F shows many rRNA coding genes, along with other protein binding genes and 73 uncharacterized genes (note that tRNAs and rRNAs were not mapped to any gene ontology term in the Entrez Gene database, they were found in EcoCyc database instead).

To narrow down the pool of the *attractor* genes and to investigate their individual function, a more stringent cut-off of expression levels (TPM) greater than 500 units at any time point, and a 3-fold change between any 2 time points unveiled 94 genes (individual function of the genes are listed in Table S5). As expected, the majority of the refined Group A (23 genes) belongs or is connected to glycolysis and fermentation. In addition, genes of aldehyde-alcohol dehydrogenase (*adhE*), transcriptional regulator (*gadE*), ferritin (*ftn*), formate hydrogenlyase regulatory protein (*hycA*) and fumarate reductase (*frdB*, *frdC*) are also present in the group.

Refined Group B contains 27 genes and mainly relates to TCA and sulphur metabolism (Table S5). Notably, it contains still several novel or unknown functional genes (*gpmA*, *osmY, ybaY*, *yfhJ*, *ytjA*). Other genes include those of entericidin B (*ecnB*), thiol peroxidase (*tpx*), chaperon protein (*hscA*) and co-chaperon protein (*hscB*), stress resistant protein (*ycfR*), and transcriptional regulators (*iscR* and *soxS*) among others.

Group C genes were not retained with the cut-off threshold, and refined Group D consists of 5 genes with various functions. Refined group E contains mostly electron transport and aerobic respiration genes, along with cytochrome *bo* oxidase genes (*cyoA*, *cyoB*, *cyoC*, *cyoD, cyoE*), biopolymer transporters (*exbB, exbD*), ornithine carbamoyltransferase (*argF, argI*), sigma factor binding protein (*crl*), peptide methionine sulfoxide reductase (*yeaA*), and alpha-ketoglutarate permease (*kgtP*). Finally, refined Group F contains 12 genes, 9 of which specifically code for ribosomal RNA (*rrlC, rrlA, rrlD, rrsH, rrlH, rrsG, rrsC, rrsE, rrfA*) (Table S5).

**Figure 7:**
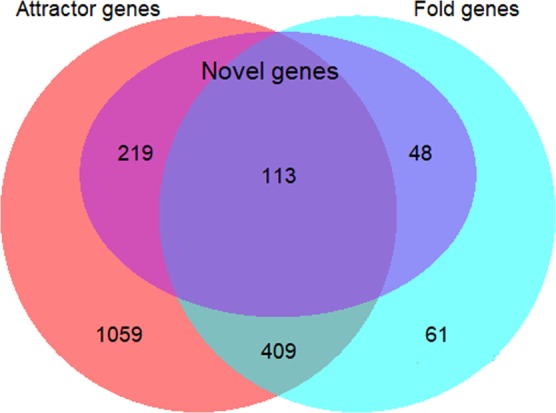
Number of *attractor* genes (red), 2-fold change genes (cyan), and novel genes (purple).

Finally, we compared our attractor analysis with the common method of choosing genes with more than 2-fold expression change between any 2 time points. This resulted in 631 genes clustered in 5 temporal groups (Fig. S6A,B). Venn diagram comparative analysis reveals 522 common genes (Fig. 7), with 48 unique novel or uncharacterized genes compared with 219 unique in the *attractor* set and 113 that are common. The notable novel genes for the 2-fold analysis include uracil permease (*yfbP*), plasmid stabilization mediating proteins (*mokC*) and a few tRNA genes (Table S6). Gene expression distribution of the *attractor* and 2-fold genes showed that the latter possess higher proportion of lowly expressed genes (Fig. S6B).

## Discussion

*E. Coli* aerobiosis is a highly investigated area of research. Although numerous works have been performed, the early dynamic transcriptome-wide response is still poorly understood. Here we investigated RNA-Seq data, consisting of 4,240 non-zero expressions, of anaerobic *E. Coli* in a bioreactor where readings were taken at 0, 0.5, 1, 2, 5 and 10 min after air supply was initiated. Unlike other approaches that typically investigated arbitrarily selected differential fold changing genes, and performed gene ontology enrichment analysis^41–43^, here we undertook multi-dimensional statistical approaches, in view of a dynamical system approach, where we queried the portion of transcriptome that are able to track the global transcriptome-wide attractor response. In other words, we investigated the spectrum of genes that are responsible for shaping the AAT.

After incorporating statistical distribution fitting (Fig. 2A,B), we retained 3,389 genes for analysis. We also included the concept of dynamical systems approach into analysing the remaining gene expressions, introducing the state space visualizations through modified linear (Pearson, *R*_*v*_) and non-linear (mutual information, *MI*_*v*_) correlation density distributions. From these, the attractor regions were generated where a subset of genes (transcriptomic elements) were shown to fall into (Fig. 3). We obtained 1800 genes that fall into the attractor basin (*attractor* genes), and another 1589 genes fall away (*non*-*attractor* genes). Notably, the *attractor* genes track the global transcriptome-wide trajectory on the PC space (Fig. 4A), and their temporal correlation analysis showed significant variation in time (Fig. 4B). Among the 1589 *non-attractor* genes, 1521 genes showed small-scaled but clear trajectory on PC space, indicating weak but collective global response, while the remaining 68 did not show any response during the AAT (Fig. S5B).

Hierarchical clustering of the *attractor* genes showed 6 temporally regulated groups (Fig. 5). Functional enrichment analysis to characterise the function of these important genes shows, as expected, glycolytic, fermentation, anaerobic respiration and cell motility related genes were gradually deactivated (Group A), whereas TCA cycle, aerobic respiration and sulphur compound metabolism were activated (Group B), inversely to Group A. These data are consistent with our general understanding of *E. coli* aerobiosis^4^. There were also several novel or uncharacterized genes significantly induced (TPM > 500 and 3-fold changes), including *osmY*, *ybaY*, *yfhJ*, and *ytjA*. Additionally, entericidin B (*ecnB*), which plays a role in bacteriolysis^44^, was also found in this group.

Group C reveals ribosomal biogenesis, translation and gene expression processes. Notably, almost 20% of the genes in this group are tRNA, along with several unknown functional genes (*yciG*, *bdm, YciG, YmdF, YobH, YciH -* Table S3). Group F, on the other hand, constitutes mainly of ribosomal RNA. The data from these 2 groups are interesting in that several recent high-profile articles have highlighted the importance of hibernating ribosomes to conserve respiration energy during anaerobic conditions, and certain enzymes “kick in” to revive the metabolism^45–49^. Also, tRNAs are key for protein synthesis and they play important roles in cellular growth, stress response and general translational regulation. The data here show several genes coding for ribosomes and tRNA perhaps play key roles in translational process that shapes anaerobic to aerobic transition.

Group D and E elucidates several hydrogenase genes which are known to be produced in anaerobic or stress conditions and participates in the reduction of fumarate and dimethyl sulfoxide (*fnr*)^50^. In addition to these,  cystathionine gama-synthase (*metB*), alkyl hydroperoxide reductase (*ahpF, ahpC*), ornithine carbamoyltransferase (*argF, argI*), and ferric iron reductase (*fhuF*) are also revealed. Many of these genes were not previously identified with the anaerobic to aerobic transition.

Overall, our work undertaking several statistical metrics, on a dynamical systems viewpoint, to infer the transcriptome-wide response of *E. Coli* aerobiosis have revealed, for the first time, the existence of a fractal portion of transcriptome (1,800 *attractor* genes) that tracks transcriptome-wide response, and is collectively crucial for the adaptive state transition. This shows a much higher resolution than the conventional 2-fold expression changing genes selected between any 2 time points (Fig. 7). Notably, previous works have mainly focused on metabolic regulatory genes, but here we show the significance of other types of genes such as tRNAs and rRNAs which are largely involved in post-transcriptional and -translational processes, in addition to 332 uncharacterised *attractor* genes. Future work could focus on elucidating the function of these genes that are captured for each temporal group of gene regulation.

## Methods

### Statistical distributions fitting

Fitting gene expression distributions was performed using the maximum-likelihood estimation method (*fitdistplus* packge^51^ for parameter estimation and the *mass* package^52^ for log-normal, Pareto, Burr, Loglogistic, Weibull and Burr distributions^53^).

### Pearson correlation

The Pearson correlation coefficient *r* between two vectors (e.g. transcriptome in two different samples), containing *n* observations (e.g. gene expression values), is defined by (for large *n*):

$$r(X,Y)=\frac{{\sum }_{i=1}^{n}({x}_{i}-{\mu }_{X})({y}_{i}-{\mu }_{Y})}{{\sigma }_{X}{\sigma }_{Y}}$$

where *x*_*i*_ and *y*_*i*_ are the *i*^*th*^ observation in the vectors ***X*** and ***Y***, respectively, *μ*_*X*_ and *μ*_*Y*_, the average values of each vector, and *σ*_*x*_ and *σ*_*y*_, the corresponding standard deviations. Pearson correlation measures linear relationship between two vectors, where *r* = 1 if the two vectors are identical, and *r* = 0 if there are no linear relationships between the vectors.

### Spearman correlation

Spearman rank correlations is defined by

$$\rho (X,Y)=1-\frac{{\sum }_{i=1}^{n}{({r}_{x,i}-{r}_{y,i})}^{2}}{n({n}^{2}-1)}$$

where *r*_*x,i*_ and *r*_*y,i*_ are the ranks of the *i*^*th*^ observation *x*_*i*_ and *y*_*i*_, in vectors ***X*** and ***Y***, respectively.

### Bicor (Biweight midcorrelation)

The biweight midcorrelation^29^ of two vectors ***X*** and ***Y*** is given by

$$bicor(X,Y)=\mathop{\sum }\limits_{i=1}^{n}{\tilde{x}}_{i}{\tilde{y}}_{i}$$

where

$${\tilde{x}}_{i}=\frac{({x}_{i}-med(x)){w}_{i}^{(x)}}{\sqrt{{\sum }_{j=1}^{n}\,{[({x}_{j}-med(x)){w}_{j}^{(x)}]}^{2}}}$$

and

$${\tilde{y}}_{i}=\frac{({y}_{i}-med(y)){w}_{i}^{(y)}}{\sqrt{{\sum }_{j=1}^{n}\,{[({y}_{j}-med(y)){w}_{j}^{(y)}]}^{2}}}$$

noting the weights for *x* or *y*, represented by general term *p*:

$${{w}_{i}}^{(p)}={\left(1-\frac{{p}_{i}-med(p)}{9mad(p)}\right)}^{2}I\left(1-|\frac{{p}_{i}-med(p)}{9mad(p)}|\right)$$

and the identity function:

$$I(x)=\{1,\,\,\,if\,x > 0\,0,\,otherwise$$

### Mutual information-based correlation MI_c_

Nonlinear dependency between two vectors ***X*** and ***Y*** can be checked by mutual information:

$$MI(X,Y)=-\mathop{\sum }\limits_{i=1}^{M}p({x}_{i})ln(p({x}_{i}))-\mathop{\sum }\limits_{i=1}^{M}p({y}_{i})ln(p({y}_{i})+\mathop{\sum }\limits_{i=1}^{M}p(x,y)ln(p(x,y))-\varepsilon $$

where^54^ the joint probability distribution function *p*(*x, y*), and marginal probability distribution functions, $p({x}_{i})$ and $p({y}_{i})$ are estimated by means of an histogram-based approach by discretizing the rank-transformed gene expression into *K* = 10 bins^18,54,55^ for *t*_*i*_ (*i* = 0,1,2..,*M*, where and *M* = 10 min). Note that systematic error *ε* occurs during the discretization, which is then subtracted from the raw *MI* value. ε is defined as minimum *MI* for 100 random permutation of the rank-transformed gene expression vector^17^. The *MI*-based correlation between ***X*** and ***Y*** is expressed via^30^:

$$M{I}_{c}={[1-{e}^{-2MI}]}^{\frac{1}{2}}$$

### Modified Pearson correlation Rv

The dynamic gene expression at time *t*_*i*_ can be defined as a *N-*dimensional vector *X*(*t*_*i*_) = (*x*_1_(*t*_*i*_), *x*_2_(*t*_*i*_), …, *x*_*N*_(*t*_*i*_)) with *x*_*j*_(*t*_*i*_) being expression value of the *j*^th^ gene at *t*_*i*_. The deviation-from-average expression vector at time *t*_*i*_ is defined as *V*(*t*_*i*_) = (*v*_1_(*t*_*i*_), *v*_2_(*t*_*i*_), …, *v*_*N*_(*t*_*i*_)) where ${v}_{j}({t}_{i})={x}_{j}({t}_{i})-\overline{{x}_{j}}$ (where$\,\overline{{x}_{j}}$ is the average expression of *j*^th^ gene over *M* + 1 time points)

The modified Pearson correlation is defined as

$${r}_{v}(V({t}_{i});\,V({t}_{0}))=\frac{V({t}_{i})\cdot V({t}_{0})}{|V({t}_{i})||V({t}_{0})|}$$

This index thus measures the temporal correlation of genome-wide expression deviations from their average values so as to allow discriminating gene expressions with different amplification but similar temporal profiles^18^.

### Modified mutual information MI_v_

Mutual information between vectors *V(t*_*i*_) and *V(t*_0_) is defined similar to formula (1) with *V(t*_*i*_) replacing ***X*** and *V(t*_0_) replacing ***Y***. The probability functions *p(x*), *p(y)* and *p(x,y)* are estimated based on discretized gene deviation data into 10 bins using histogram-based approach. Systematic error ε is defined as minimum *MI* for 100 random permutation of gene deviation vectors *V(t*_*i*_*)*^18^. Finally, to compare *MI* among different replicates, we used the normalized value:

$$M{I}_{v}(V({t}_{i}),V({t}_{0}))=\frac{MI(V({t}_{i}),V({t}_{0}))}{MI(V({t}_{0}),V({t}_{0}))}$$

### Ranking genome elements

The whole transcriptome (3389 genes) was sorted according to their standard deviation across 6 time points, from the highest to the lowest: $\sigma ={\cup }_{j=1}^{N}{\sigma }_{j}(N=3389)$, with ${\sigma }_{j}=\sqrt{\frac{1}{M+1}\mathop{\sum }\limits_{i=0}^{M}{({x}_{j}({t}_{i}))}^{2}}(M=6)$ being the standard deviation of gene *j*^*th*^ expression across 6 time points. After that, we divided the ranked standard deviation vector $\sigma$ into *p* groups, each group with *n* genes (*n* = 25, 50, 100, 200, 400, 600, 800, 1000). Note that we choose *p* = ⌈$\frac{N}{n}$⌉, the *p*^th^ group contained *n* genes which can be overlapped with the (*p* − 1)^th^ group. Next, we examined the trajectory on *MI*_*v*_ *− R*_*v*_ space for each individual group of genes to check whether it fall into attractor region.

### Determination of attractor region on R_v_-MI_v_ space

Attractor boundary was defined on the superimposed probability density (SPD) distribution of modified Pearson correlation *R*_*v*_ and modified mutual information *MI*_*v*_ for whole genome (3389 genes). Distribution of whole genome *R*_*v*_ and *MI*_*v*_ was generated by randomly choosing *n* = 100 genes for 100 times from the pool of 3389 genes, and SPD of these 100 repeats was estimated on discretised lattice by 2D kernel density estimation using the *mass* library in R programming^52^.

Attractor boundary was determined by the inflection points on the SPD of whole genome *R*_*v*_ and *MI*_*v*_, where the inflection points^18^ were determined as highest gradients in vertical and horizontal directions from the local points on the lattice. Averaging the z-coordinates of the vertical and horizontal inflection points determine the z-coordinate of inflection curve, or attractor boundary contour.

### Hierarchical clustering

Hierarchical clustering was performed on normalized expressions of *attractor* and *pseudo-attractor* genes using Ward clustering method^56^. Normalized expression of the *j*^th^ gene at time *t*_*i*_ is defined as^17^ ${z}_{j}({t}_{i})=({x}_{j}({t}_{i})-\overline{{x}_{j}})/{\sigma }_{j}$ where ${x}_{j}({t}_{i})$ is expression of the *j*^th^ gene at time *t*_*i*_, $\overline{{x}_{j}}$ is the mean expression across all time points, and ${\sigma }_{j}$ is the standard deviation. As a result, 9 clusters were obtained, which were further regrouped according to 5 distinct temporal average expression patterns for *attractor* genes, and 4 distinct temporal average expression patterns for *pseudo-attractor* genes.

## Supplementary information

Supplementary Information.

Table S2.

Table S3.

Table S4.

Table S5.

Table S6.

## Data Availability

The R-codes for transcriptomics analysis are available from the authors upon request. The *E. coli* data is obtained using GEO accession number GSE71562.
