# Supplementary material for: Attractor Concepts to Evaluate the Transcriptome-wide Dynamics Guiding Anaerobic to Aerobic State Transition in Escherichia coli
Source: Sci Rep. 2020 Apr 3;10:5878. doi: 10.1038/s41598-020-62804-3 (PMC7125300; doi:10.1038/s41598-020-62804-3)
Supplement: Supplementary file 1 — Supplementary Information. [file 41598_2020_62804_MOESM1_ESM.pdf]

Supplementary Figures and Tables

Attractor Concepts to Evaluate the Transcriptome-wide Dynamics Guiding Anaerobic to Aerobic State Transition in *Escherichia coli*

Thuy Tien Bui[1] and Kumar Selvarajoo[1,2*]

[1]Singapore Institute of Food and Biotechnology Innovation (SIFBI), Agency for Science, Technology and Research A*STAR, Proteos, 61 Biopolis Drive, Singapore 138673, Singapore.

[2]Synthetic Biology for Clinical and Technological Innovation (SynCTI),

National University of Singapore, 28 Medical Drive, Singapore 117456, Singapore

*Corresponding author email: kumar_selvarajoo@biotrans.a-star.edu.sg

Supplemental figure legend

***Figure S1*. Comparison transcriptome-wide data with statistical distributions for all three replicates (denoted as sample a, b, and c) at all time (0, 0.5, 1, 2, 5, 10 minutes). (A)** Cumulative distribution functions versus TPM in logscale, and **(B)** Quantile-quantile plot between transcriptome data (experimental data – black colour) and lognormal (red), Pareto (light green), Burr (cyan), loglogistic (blue), Weibull (purple), and gamma (grey) statistical distributions.

***Figure S2*. Transcriptome-wide correlation in time. (A)** Spearman correlation and **(B)** Biweight midcorrelation (see Methods) between time t_i (0, 0.5, 1, 2, 5, 10 min) and t_0 (0 min) of all 3 replicates (replicate a – red, replicate b – green, replicate c - blue) across all 3391 genes with expression above 5 TPM (left panel), and across 3389 genes (2 highest expressed genes, *rnpB* and *lpp*, were removed – right panel). Smooth decreasing trend with time is observed for all correlation metrics used.

***Figure S3*. Properties of *attractor* and *non-attractor* genes. (A)** Distribution and **(B)** Standard deviations of R_v (top panel) and MI_v (bottom panel) with different genome element size (denoted as n) of all replicates (denoted as sample a, b, and c) at time t = 0.5, 1 2, 5, 10 minutes. Standard deviation of R_v and MI_v decreases as n increases, following the law of large number (black). **(C)** Euclidian distance (the summed difference across all time points) of 100-gene transcriptomic elements compared with whole transcriptome trajectory. The elements are categorized into 4 types: falling above attractor region

(empty triangle symbol), falling into attractor region (black filled circle symbol), falling out of attractor region but having close distance to whole transcriptome trajectory (*pseudo-attractor* - empty circle symbol), and falling out of attractor region (*non-attractor* – empty square symbol). **(C)** Trajectories of elements falling above and into attractor (500 genes – black), *pseudo-attractor* elements (1300 genes - green), and *non-attractor* elements (1589 genes - blue) overlaid on SPD of whole transcriptome R_v and MI_v. Trajectory for each of the three sets of gene was generated by averaging 100 trajectories of 100 randomly chosen genes from the respective pool of genes. (**D**). Kernel density of expression level for *attractor* (red), and *non-attractor* (green) sets of gene at time t = 0, 0.5, 1, 2, 5, 10 minutes. Kernel density was estimated on the averaged expression of 3 replicates.

***Figure S4*. Trajectory of genome elements with element size *n* = 100 genes overlaid on superimposition of probability distribution (SPD) of whole-genome.** R_v and MI_v values of each 100-gene element at time t = 0, 0.5, 1, 2, 5, 10 minutes formed their trajectory, with the transcriptomic elements sorted by **(A)** standard deviation of individual gene expression in the whole transcriptome (3389 genes) across 6 time points, giving 34 elements and **(B)** maximum fold-change of individual gene expression across all time points. Note that trajectory for each set of gene was obtained by averaging the corresponding trajectories of 3 replicates.

Figure S5. *Attractor* and *non-attractor* genes trajectory on PC space and their temporal correlation (A) Proportion of variance explained by the first 6 principal components in *whole transcriptome* (top left panel), *attractor* (top right panel), *pseudo-attractor* (bottom left panel), and *non-attractor* (bottom right panel) sets of gene. PC1 and PC2 account for more than 70% variance. (**B**) Temporal correlation of *whole transcriptome* (black), *attractor* (red), *collective non-attractor* (blue) and *no response* (brown) genes using Pearson (top left panel), Spearman (top right panel), Biweight midcorrelation (bottom left panel) and Mutual Information-based (bottom right panel) correlation metrics. Removing 68 *no response* genes from the 1589 *non-attractor* genes did not affect the *non-attractor* trajectory and temporal correlation profile.

Figure S6. Properties of genes with two-fold expression change in comparison with *attractor* genes (A) – (B) Major gene expression patterns of 631 *2-fold change* genes. (**A**): Hierarchical clustering of *attractor* genes reveals 12 clusters of temporal expression profiles. (**B**) Five temporal average expression profile constructed by regrouping the 12 clusters: Group A: Gradual decay: Group B: Gradual activation; Group C: Fast activation, followed by decay and re-activation; Group D: Early activation, followed by decay; Group E: Early decay, followed by activation. **(C)** Kernel density of expression level for *attractor* (red), and *2-fold change* (blue) sets of gene at time t = 0, 0.5, 1, 2, 5, 10 minutes. Kernel density was estimated on the averaged expression of 3 replicates.

Figure S1A

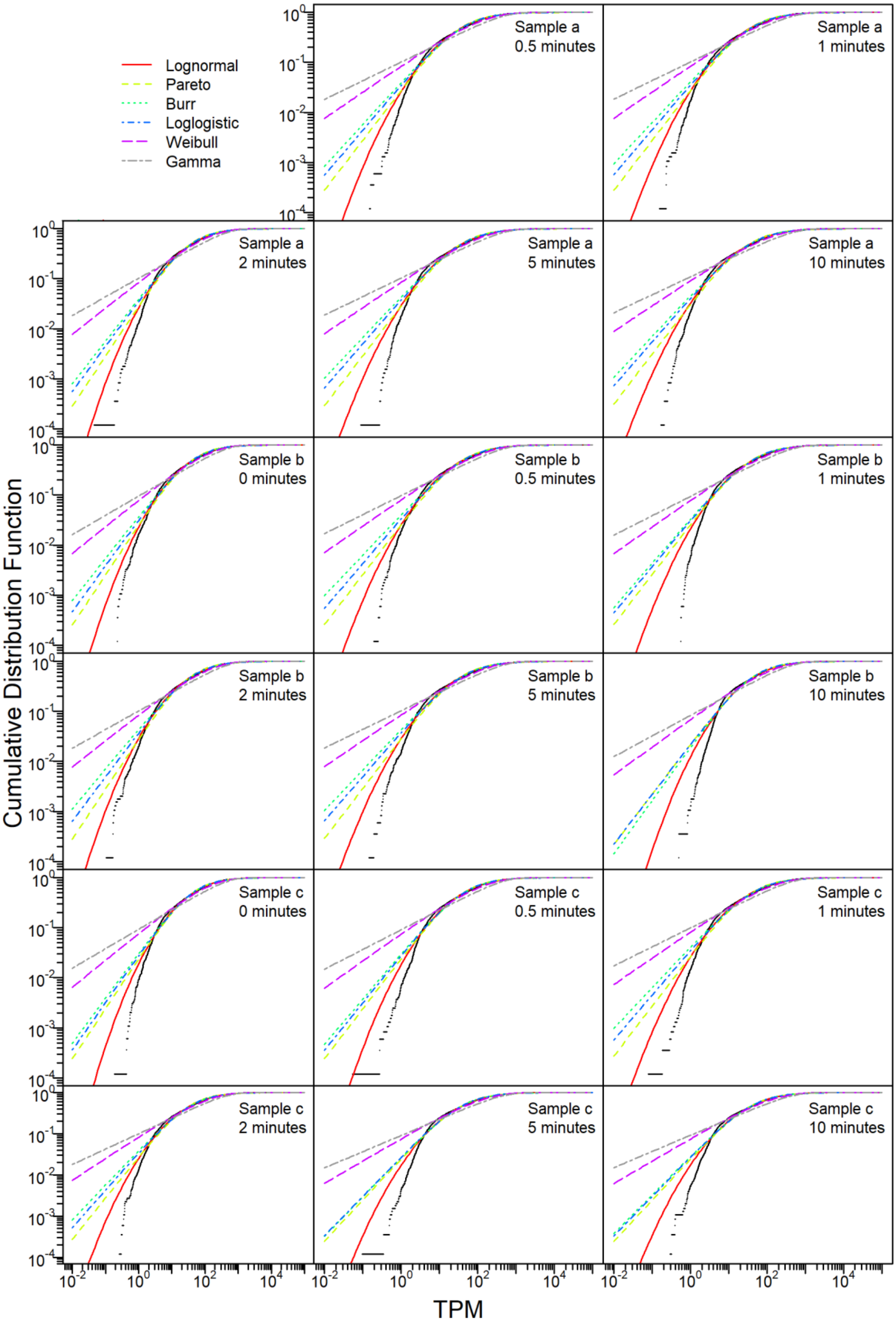

Lognormal
Pareto
Burr
Loglogistic
Weibull
Gamma
Sample a 0.5 minutes
Sample a 1 minutes
Sample a 2 minutes
Sample a 5 minutes
Sample a 10 minutes
Sample b 0 minutes
Sample b 0.5 minutes
Sample b 1 minutes
Sample b 2 minutes
Sample b 5 minutes
Sample b 10 minutes
Sample c 0 minutes
Sample c 0.5 minutes
Sample c 1 minutes
Sample c 2 minutes
Sample c 5 minutes
Sample c 10 minutes
Cumulative Distribution Function
TPM

Figure S1B

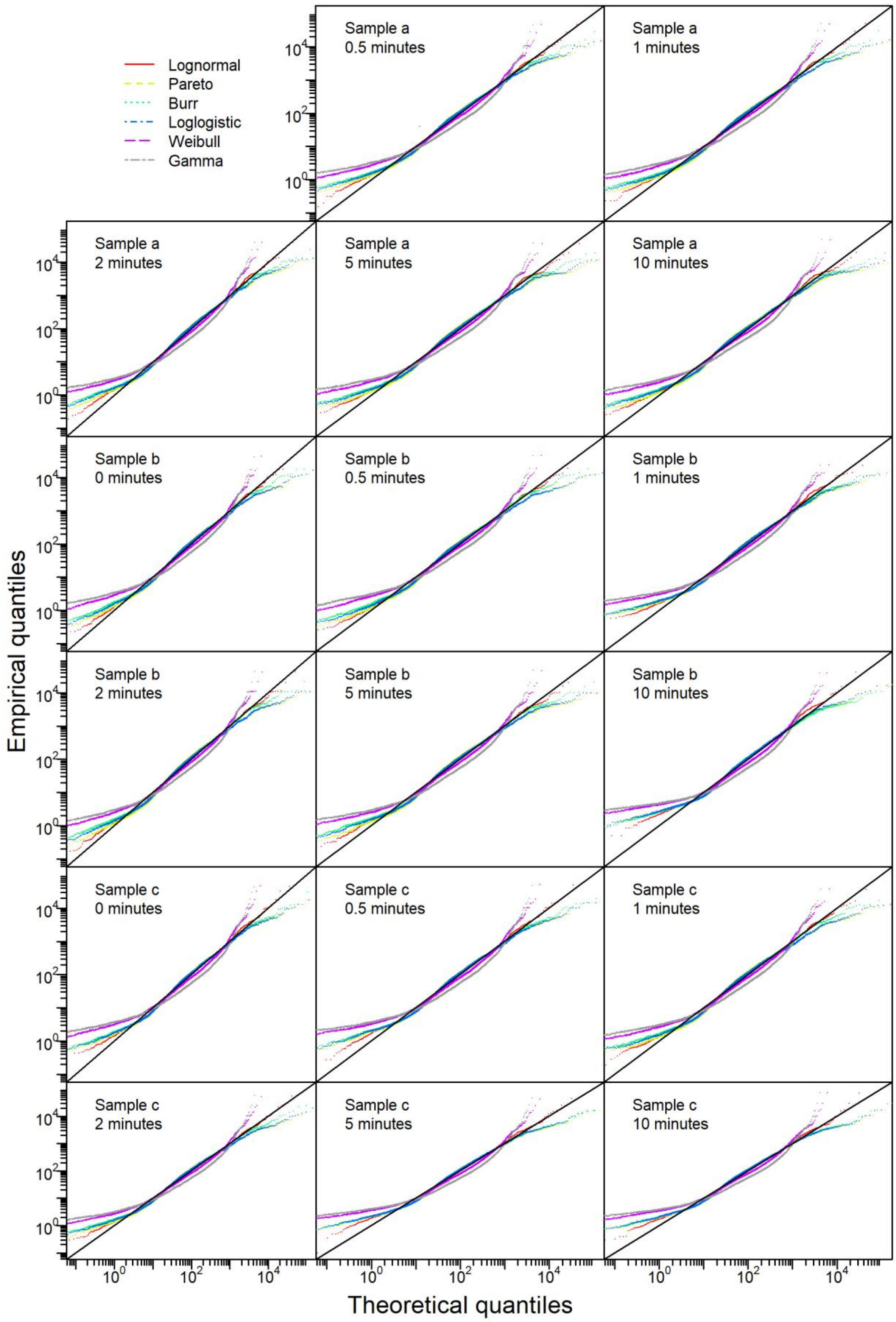

Lognormal
Pareto
Burr
Loglogistic
Weibull
Gamma
Sample a
0.5 minutes
Sample a
1 minutes
Sample a
2 minutes
Sample a
5 minutes
Sample a
10 minutes
Sample b
0 minutes
Sample b
0.5 minutes
Sample b
1 minutes
Sample b
2 minutes
Sample b
5 minutes
Sample b
10 minutes
Sample c
0 minutes
Sample c
0.5 minutes
Sample c
1 minutes
Sample c
2 minutes
Sample c
5 minutes
Sample c
10 minutes
Empirical quantiles
Theoretical quantiles

Figure S2

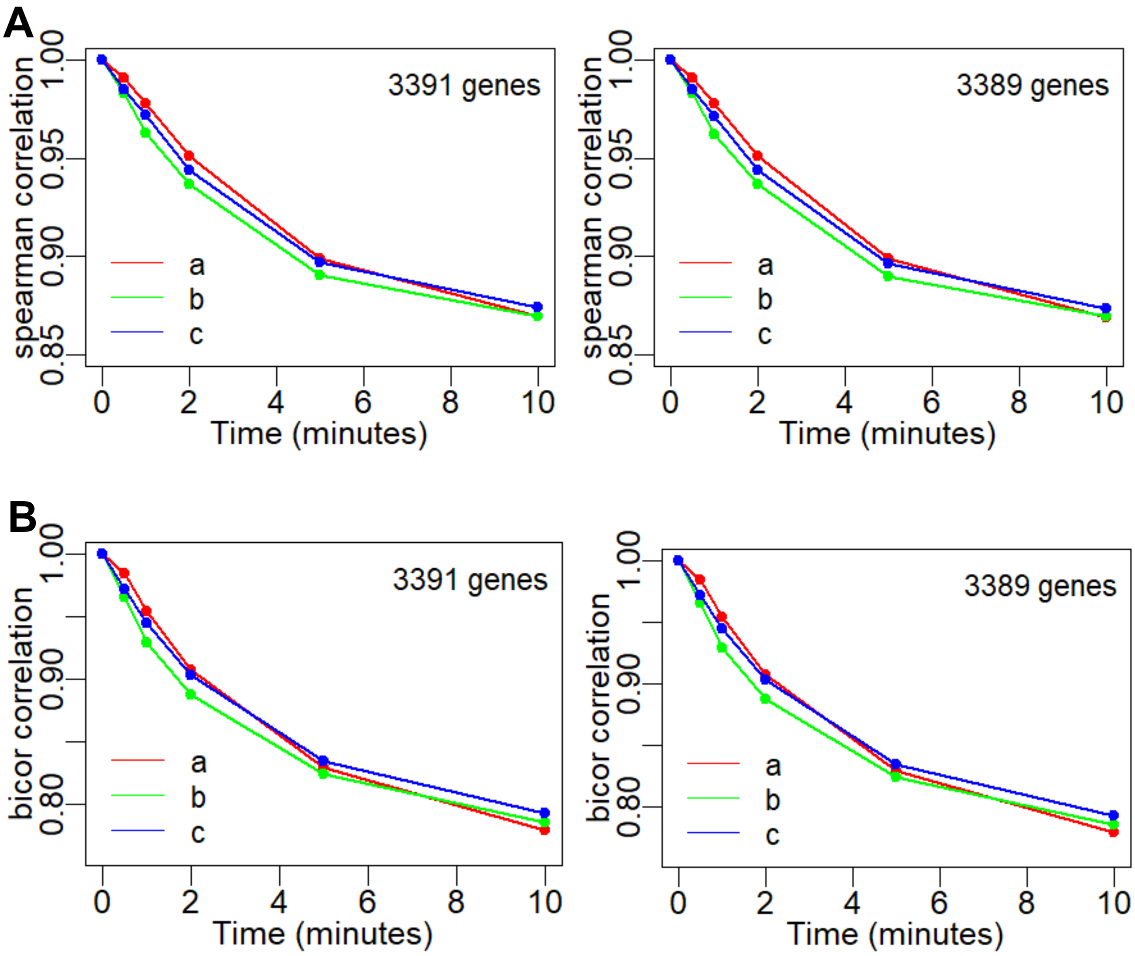
A
spearman correlation
0.85
0.90
0.95
1.00
3391 genes
a
b
c
0 2 4 6 8 10
Time (minutes)
spearman correlation
3389 genes
a
b
c
Time (minutes)
B
bicor correlation
0.80
0.90
1.00
3391 genes
a
b
c
Time (minutes)
bicor correlation
3389 genes
a
b
c
Time (minutes)

Figure S3A

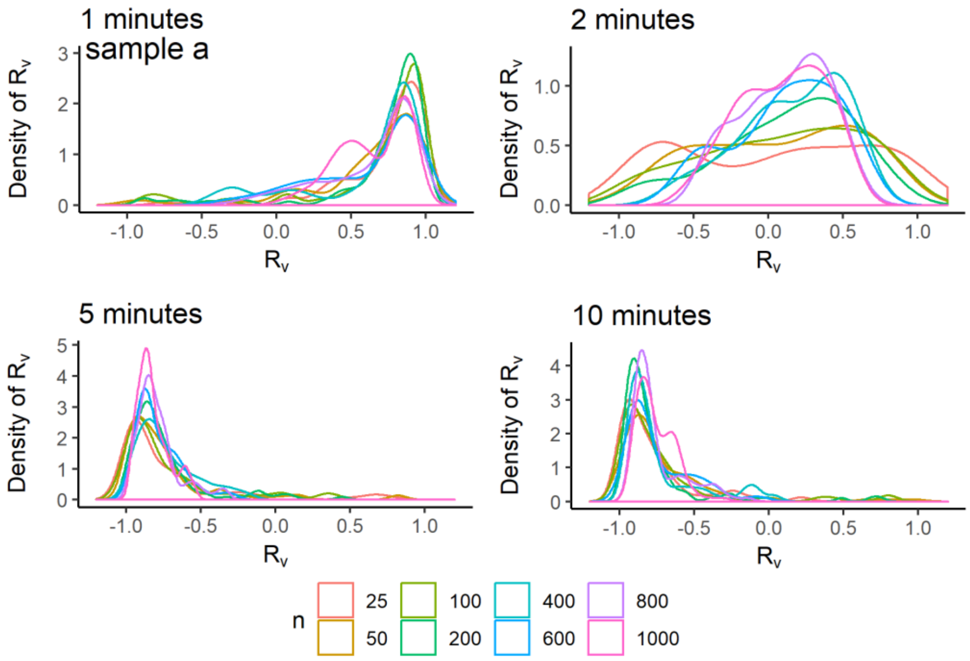
1 minutes
sample a
2 minutes
5 minutes
10 minutes
Density of R_v
R_v
n
25
50
100
200
400
600
800
1000

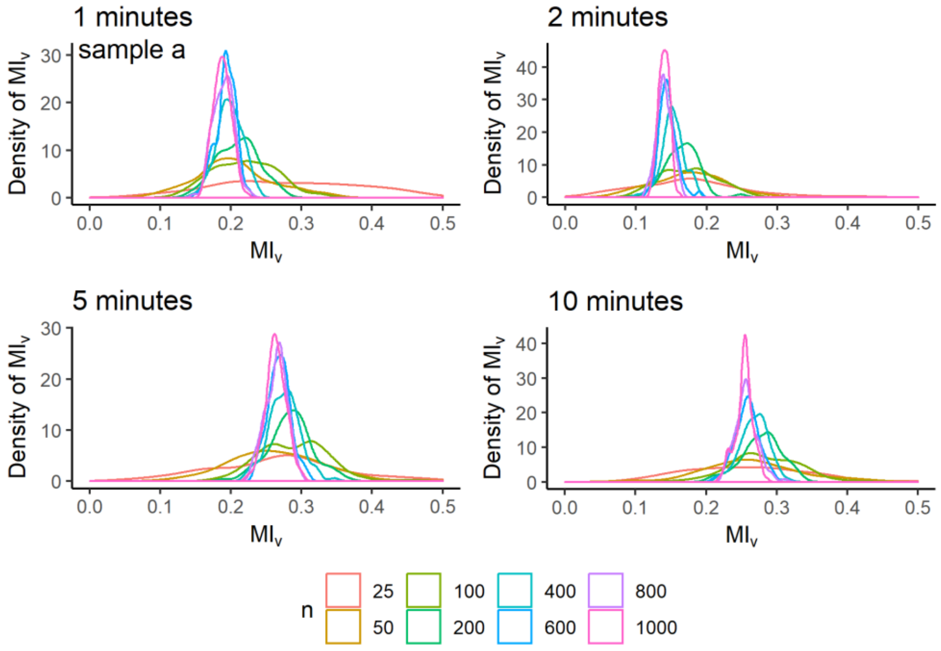
1 minutes
sample a
2 minutes
5 minutes
10 minutes
Density of MI_v
MI_v
n
25
50
100
200
400
600
800
1000

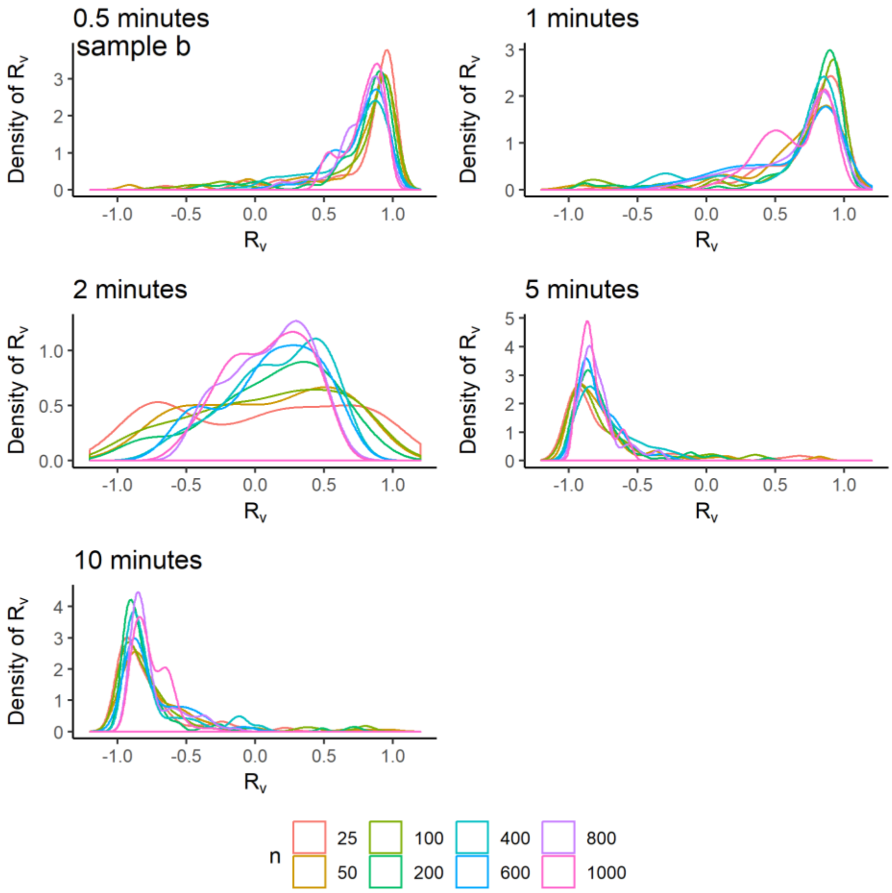
0.5 minutes
sample b
1 minutes
2 minutes
5 minutes
10 minutes
Density of R_v
R_v
n
25
50
100
200
400
600
800
1000

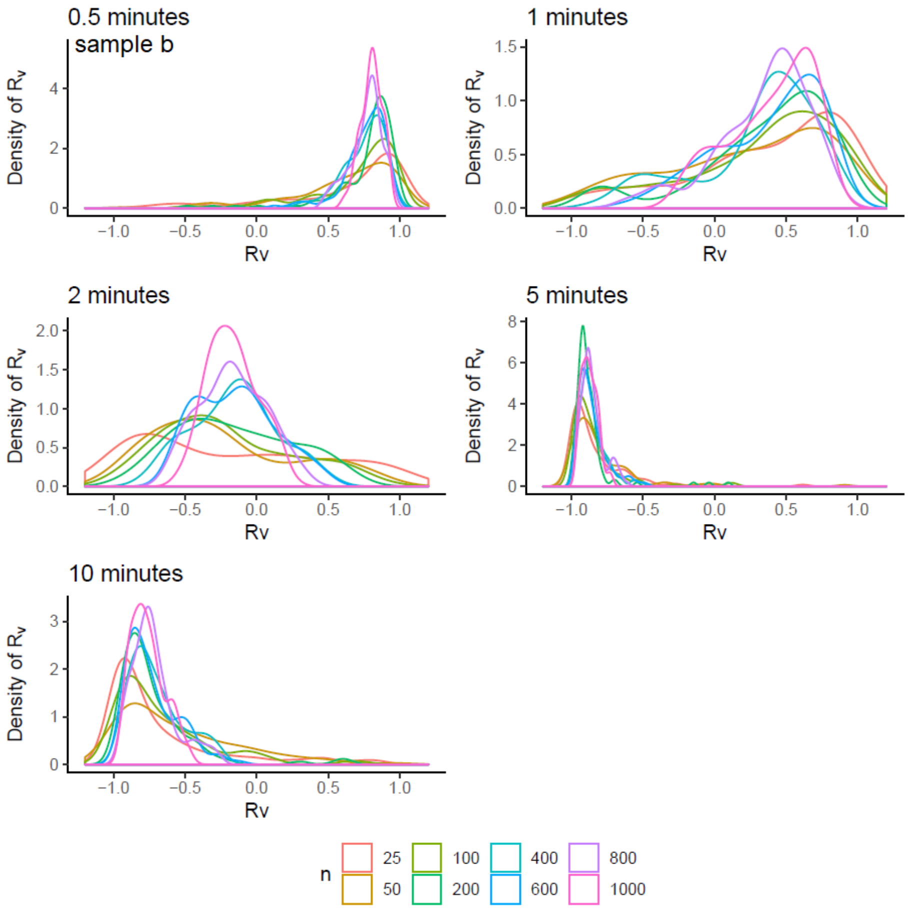
0.5 minutes
sample b
1 minutes
2 minutes
5 minutes
10 minutes
Density of R_v
Rv
n
25
50
100
200
400
600
800
1000

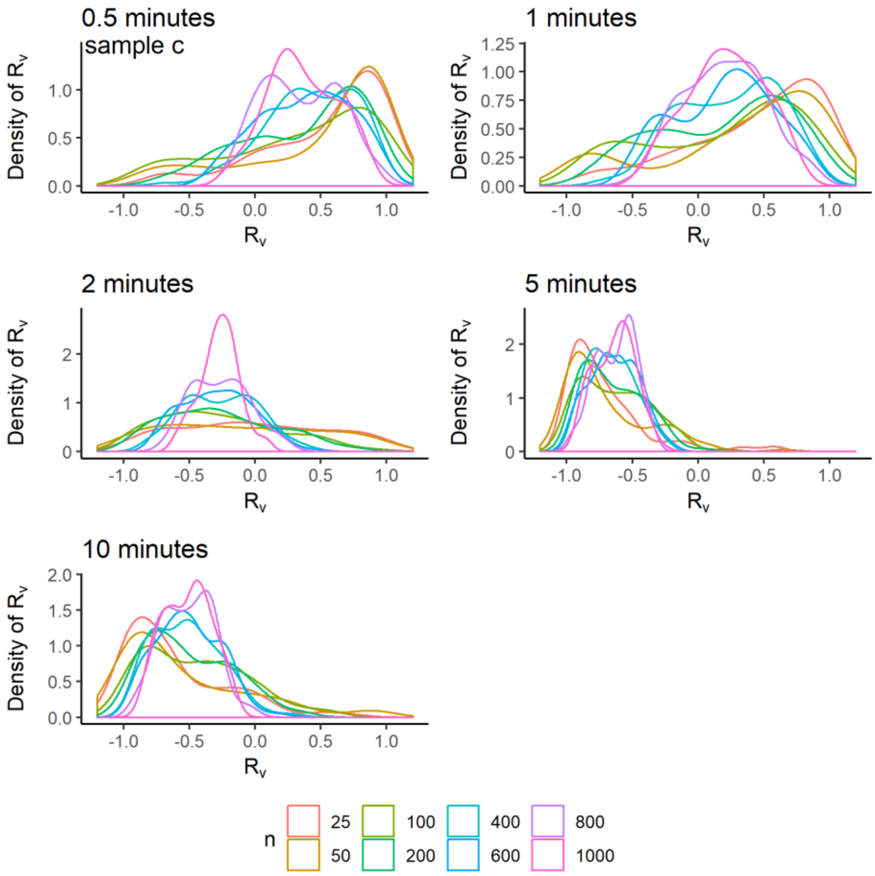
0.5 minutes
sample c
1 minutes
2 minutes
5 minutes
10 minutes
Density of R_v
R_v
-1.0
-0.5
0.0
0.5
1.0
n
25
50
100
200
400
600
800
1000

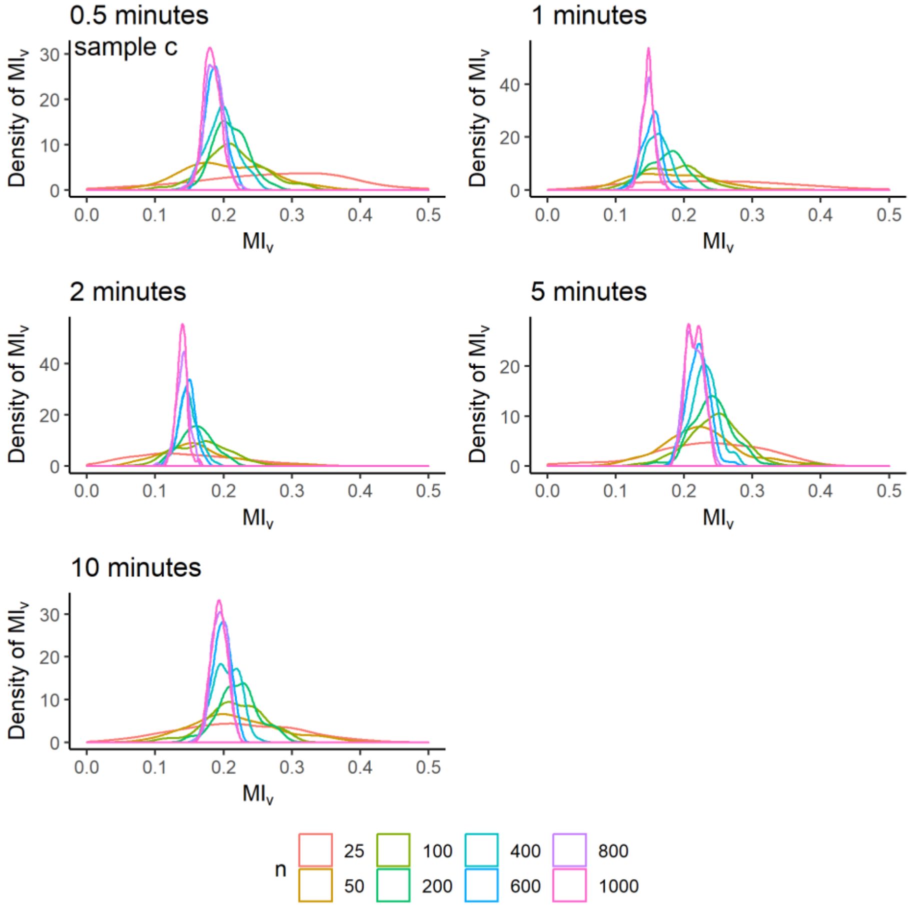
0.5 minutes
sample c
1 minutes
2 minutes
5 minutes
10 minutes
Density of MI_v
MI_v
0.0
0.1
0.2
0.3
0.4
0.5
n
25
50
100
200
400
600
800
1000

Figure S3B

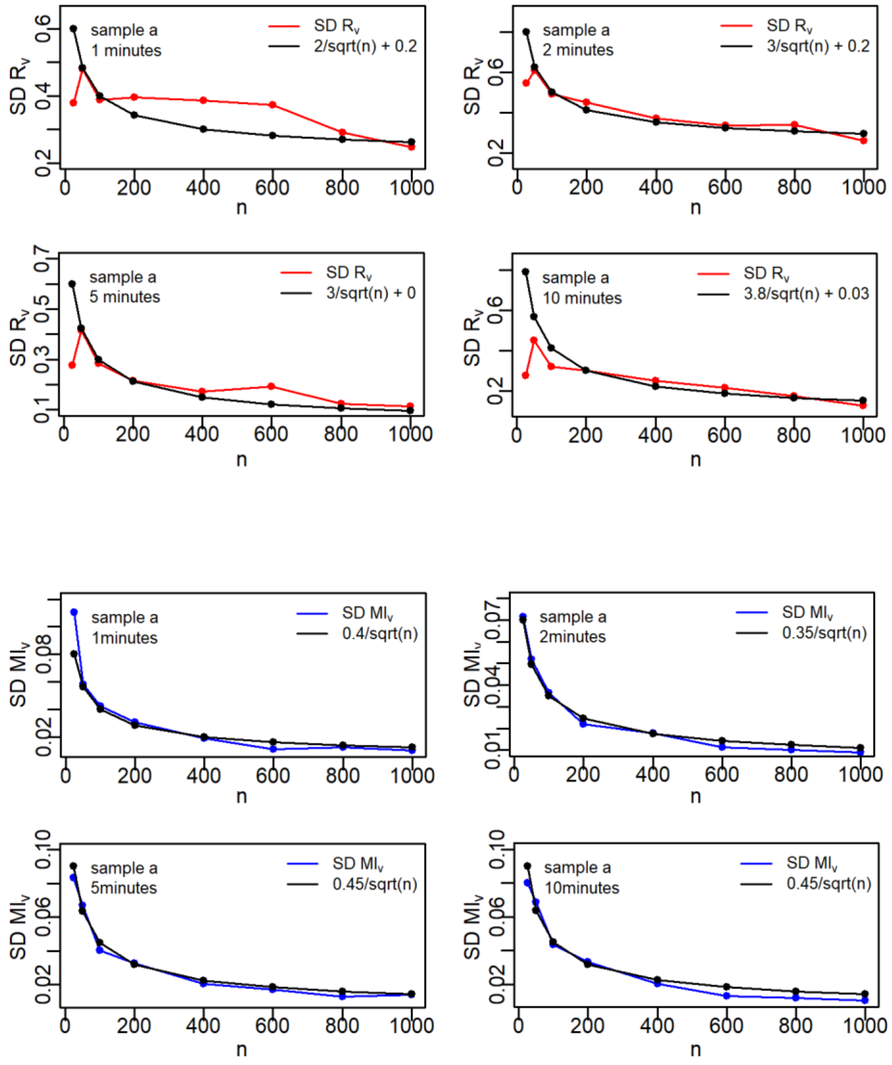
sample a
1 minutes
SD R_v
2/sqrt(n) + 0.2
sample a
2 minutes
SD R_v
3/sqrt(n) + 0.2
sample a
5 minutes
SD R_v
3/sqrt(n) + 0
sample a
10 minutes
SD R_v
3.8/sqrt(n) + 0.03
sample a
1minutes
SD MI_v
0.4/sqrt(n)
sample a
2minutes
SD MI_v
0.35/sqrt(n)
sample a
5minutes
SD MI_v
0.45/sqrt(n)
sample a
10minutes
SD MI_v
0.45/sqrt(n)
n

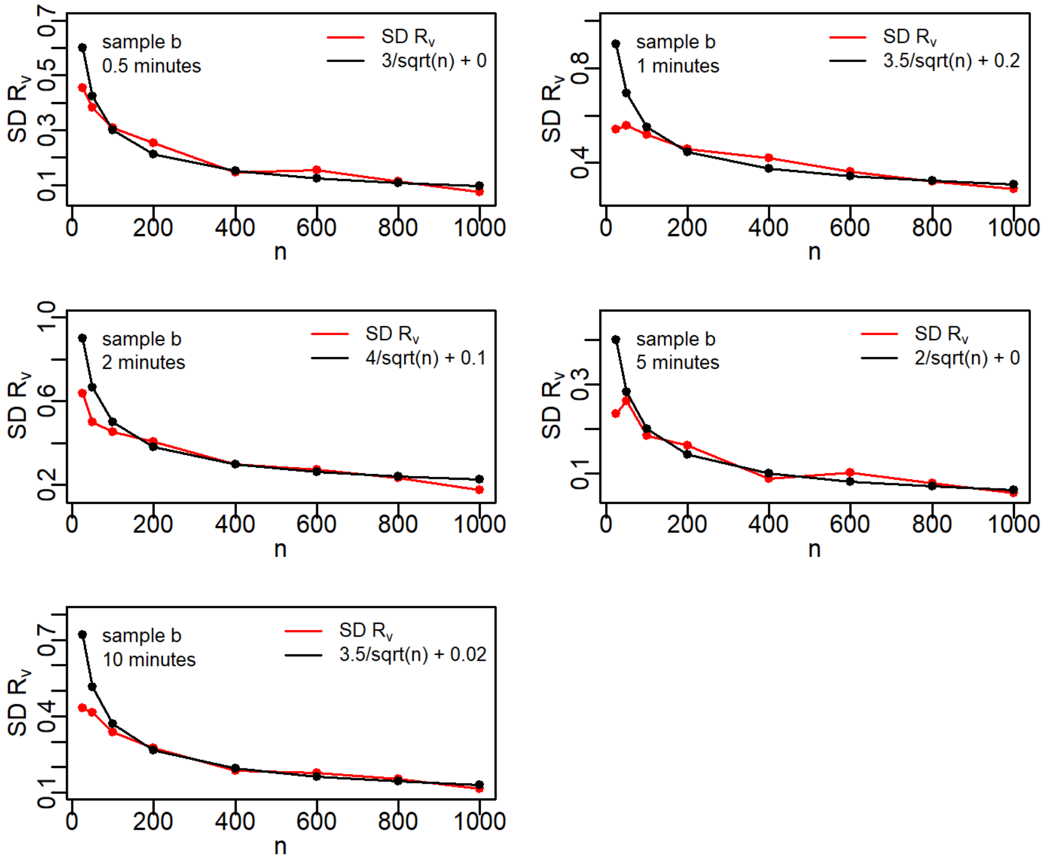
sample b
0.5 minutes
SD Rv
3/sqrt(n) + 0
sample b
1 minutes
SD Rv
3.5/sqrt(n) + 0.2
sample b
2 minutes
SD Rv
4/sqrt(n) + 0.1
sample b
5 minutes
SD Rv
2/sqrt(n) + 0
sample b
10 minutes
SD Rv
3.5/sqrt(n) + 0.02
SD Rv
n

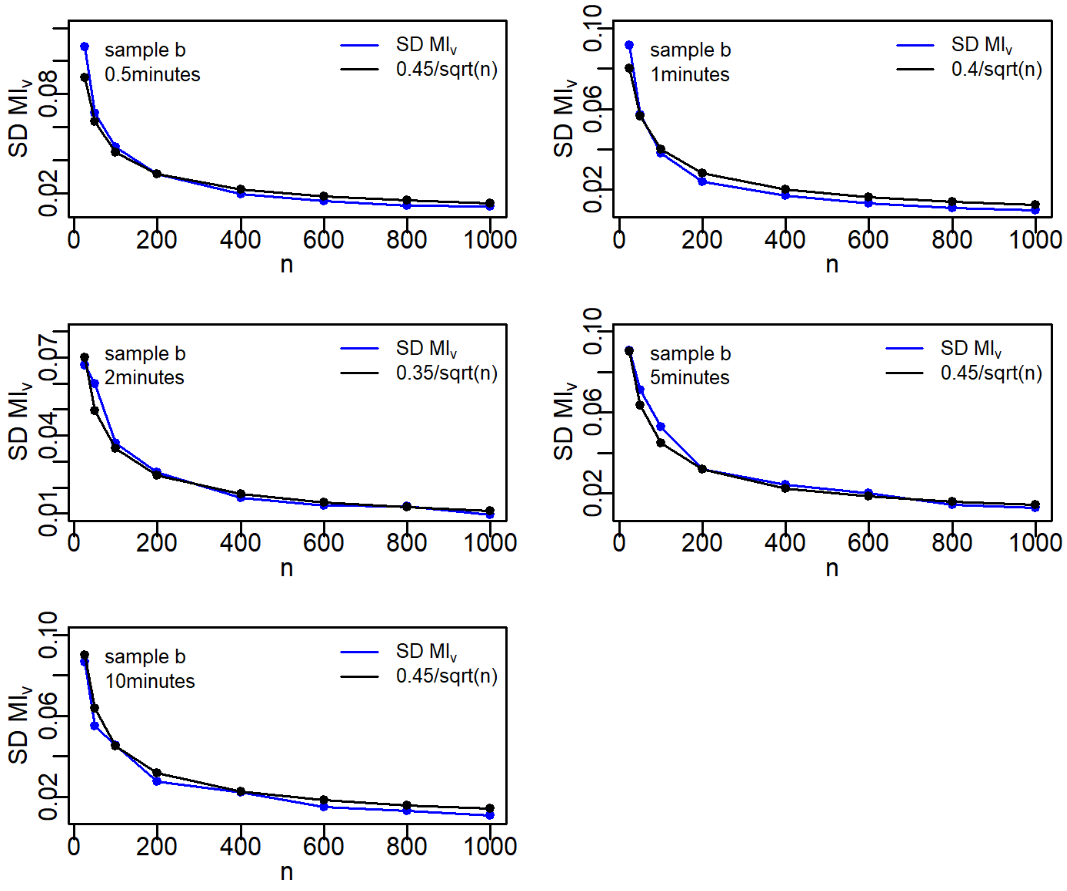
sample b
0.5minutes
SD MIv
0.45/sqrt(n)
sample b
1minutes
SD MIv
0.4/sqrt(n)
sample b
2minutes
SD MIv
0.35/sqrt(n)
sample b
5minutes
SD MIv
0.45/sqrt(n)
sample b
10minutes
SD MIv
0.45/sqrt(n)
SD MIv
n

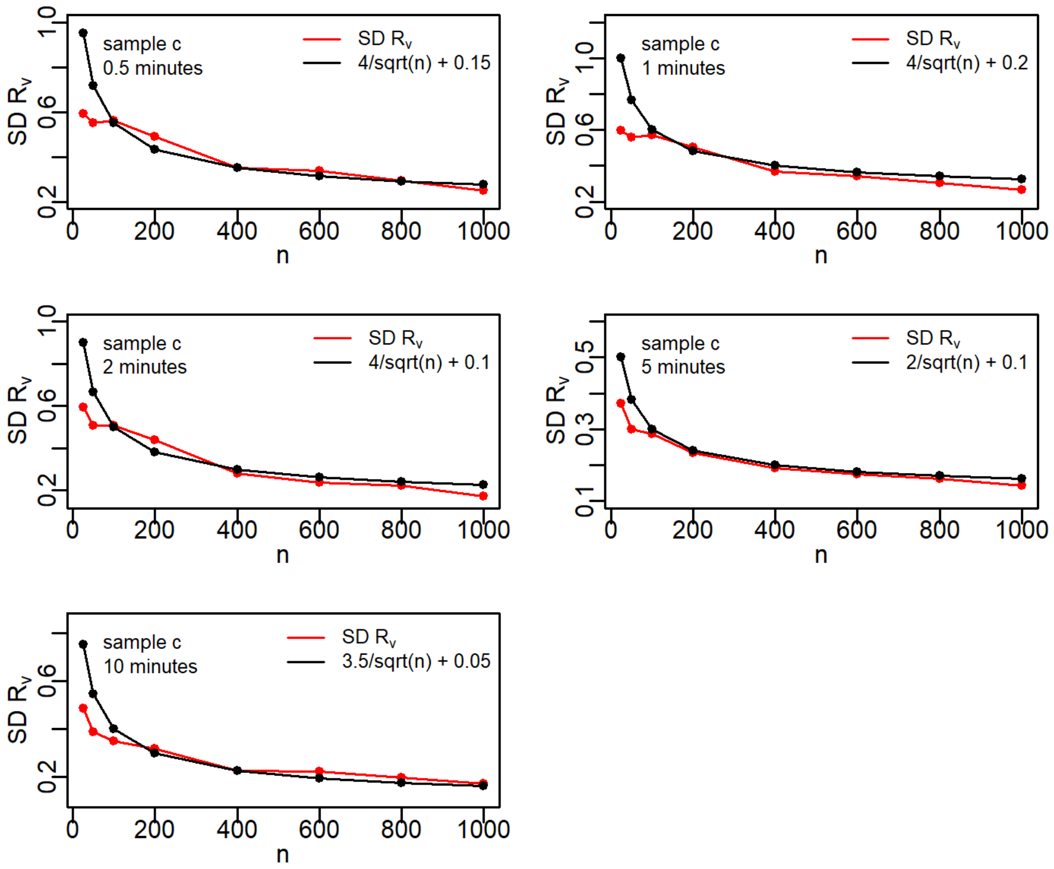

sample c
0.5 minutes
SD R_v
4/sqrt(n) + 0.15
sample c
1 minutes
SD R_v
4/sqrt(n) + 0.2
sample c
2 minutes
SD R_v
4/sqrt(n) + 0.1
sample c
5 minutes
SD R_v
2/sqrt(n) + 0.1
sample c
10 minutes
SD R_v
3.5/sqrt(n) + 0.05
n

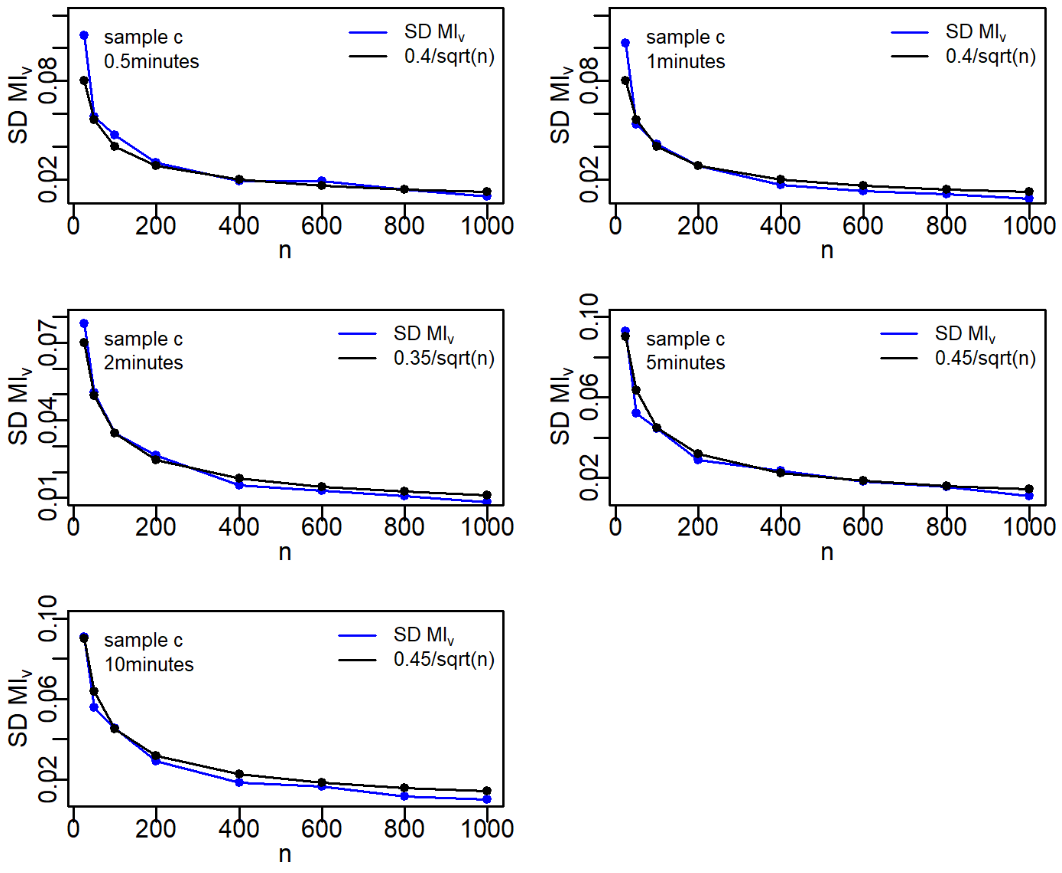

sample c
0.5minutes
SD MI_v
0.4/sqrt(n)
sample c
1minutes
SD MI_v
0.4/sqrt(n)
sample c
2minutes
SD MI_v
0.35/sqrt(n)
sample c
5minutes
SD MI_v
0.45/sqrt(n)
sample c
10minutes
SD MI_v
0.45/sqrt(n)
n

Figure S3C

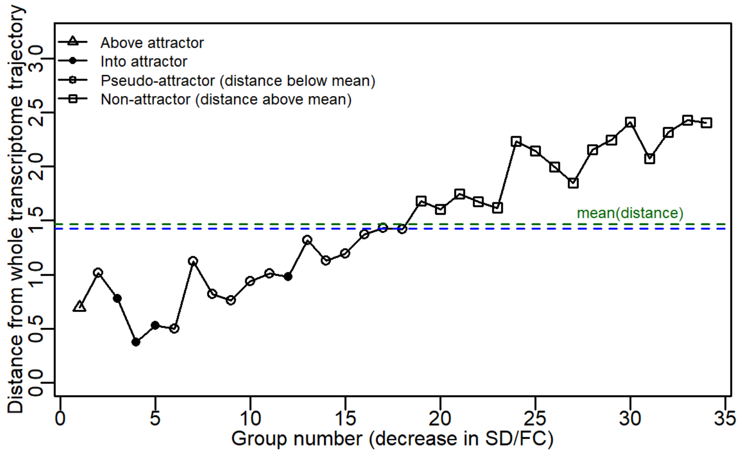

Figure S3D

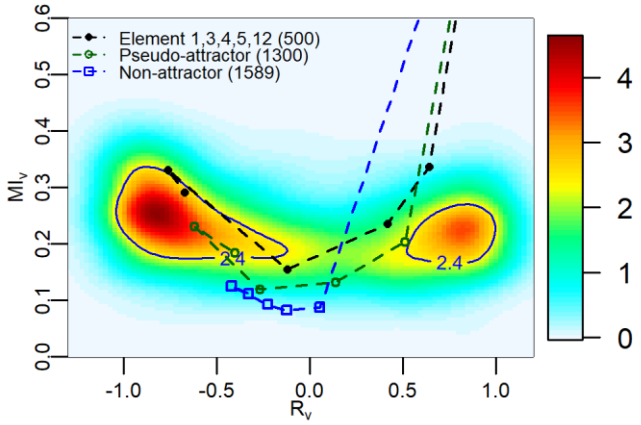

Figure S3E

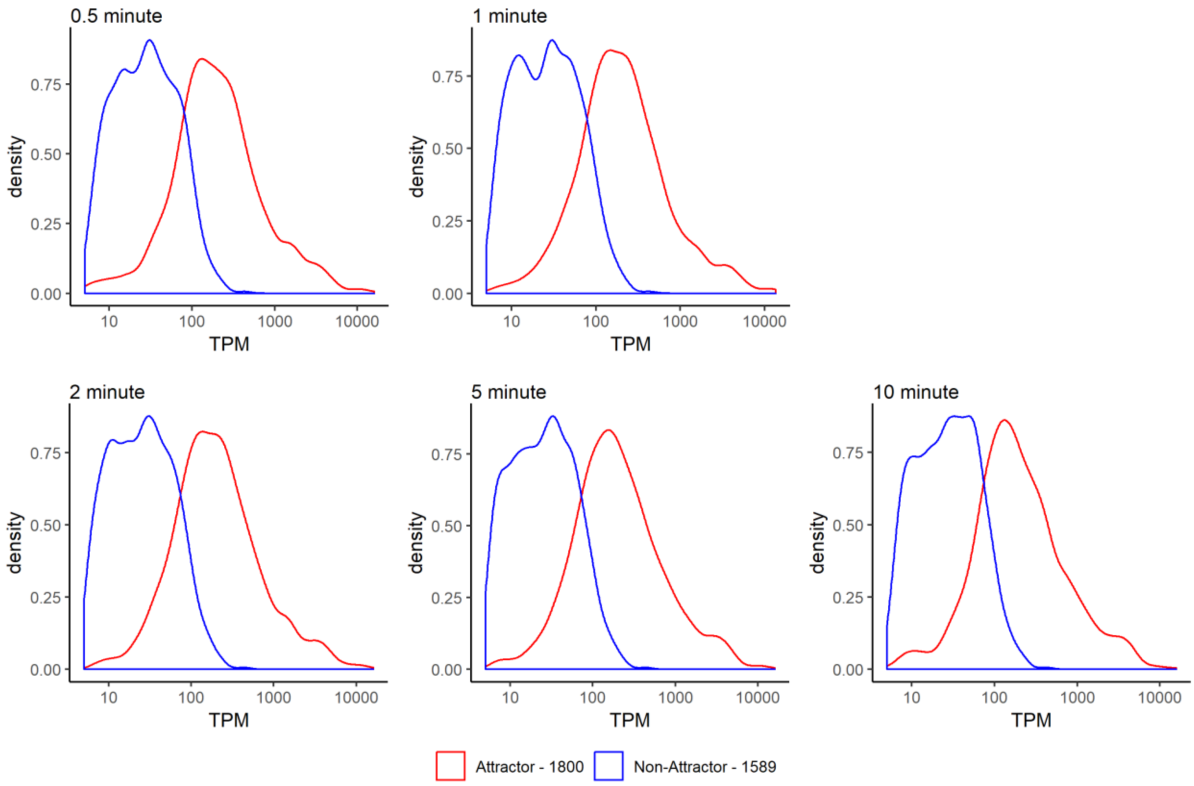

0.5 minute
1 minute
2 minute
5 minute
10 minute
density
0.00
0.25
0.50
0.75
10
100
1000
10000
TPM
Attractor - 1800
Non-Attractor - 1589

Figure S4A

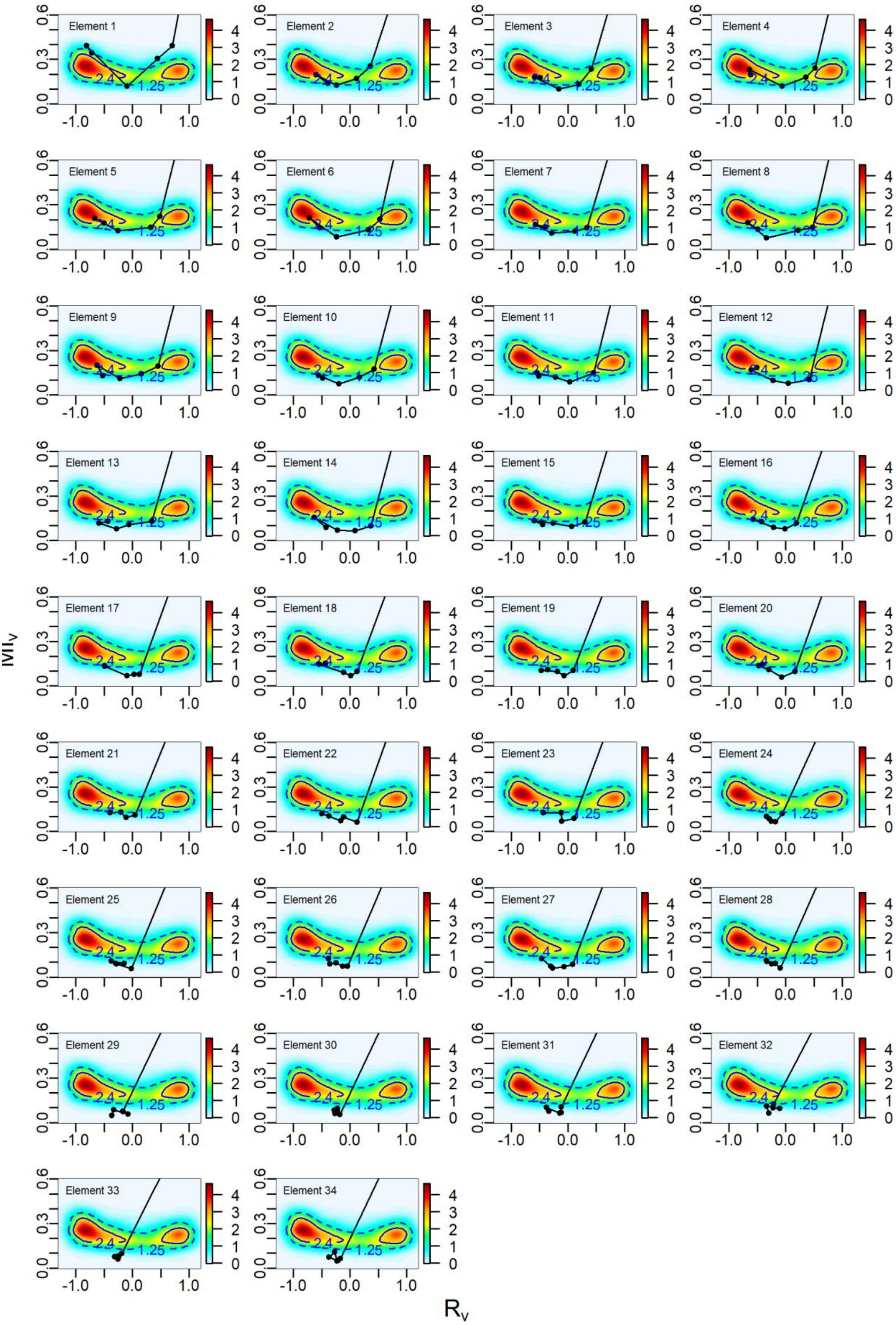
Element 1
Element 2
Element 3
Element 4
Element 5
Element 6
Element 7
Element 8
Element 9
Element 10
Element 11
Element 12
Element 13
Element 14
Element 15
Element 16
Element 17
Element 18
Element 19
Element 20
Element 21
Element 22
Element 23
Element 24
Element 25
Element 26
Element 27
Element 28
Element 29
Element 30
Element 31
Element 32
Element 33
Element 34
2.4
1.25
-1.0
0.0
1.0
0.0
0.3
0.6
$|\Pi|_V$
R_V

Figure S4B

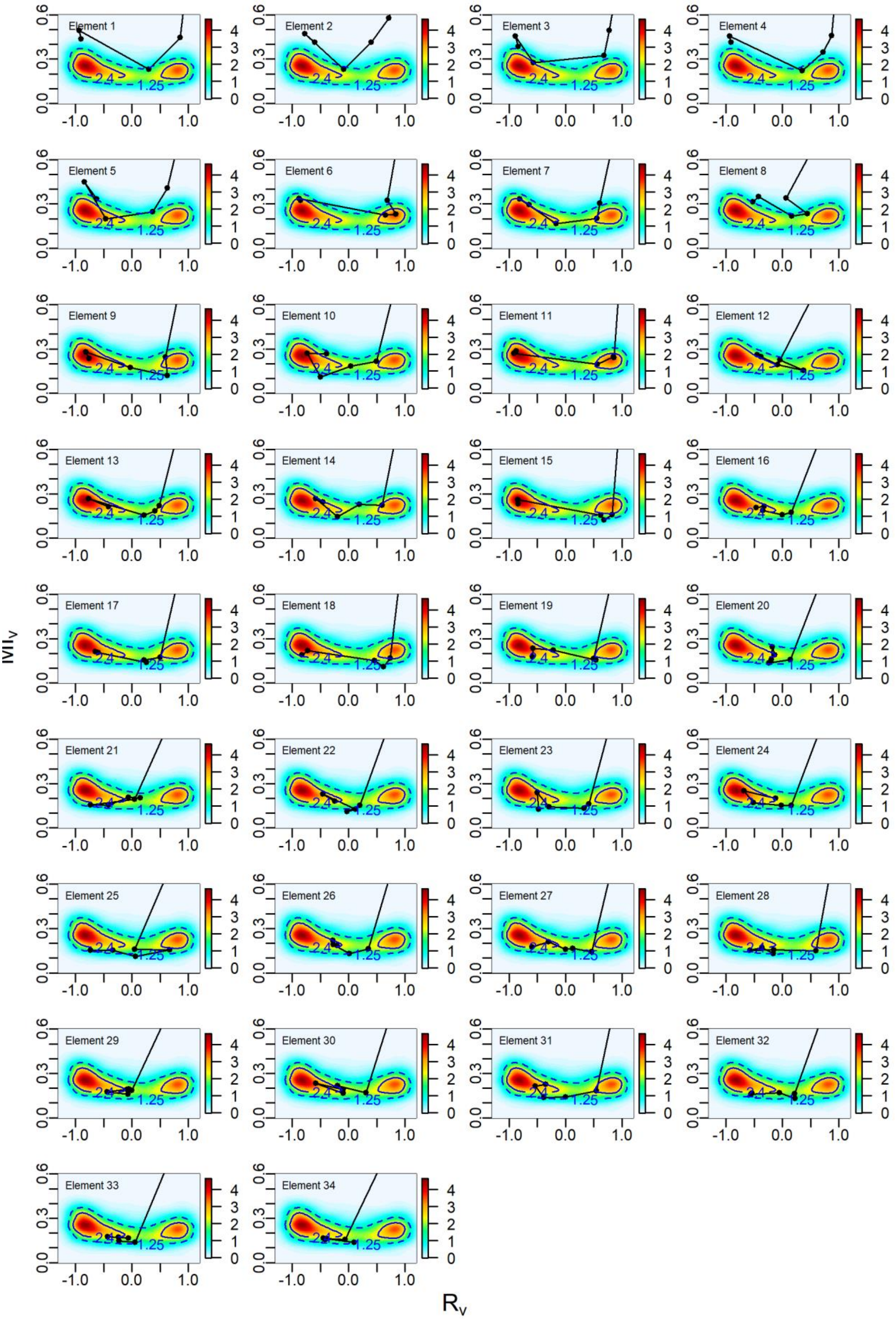
Element 1
Element 2
Element 3
Element 4
Element 5
Element 6
Element 7
Element 8
Element 9
Element 10
Element 11
Element 12
Element 13
Element 14
Element 15
Element 16
Element 17
Element 18
Element 19
Element 20
Element 21
Element 22
Element 23
Element 24
Element 25
Element 26
Element 27
Element 28
Element 29
Element 30
Element 31
Element 32
Element 33
Element 34
2.4
1.25
R_v

Figure S5A

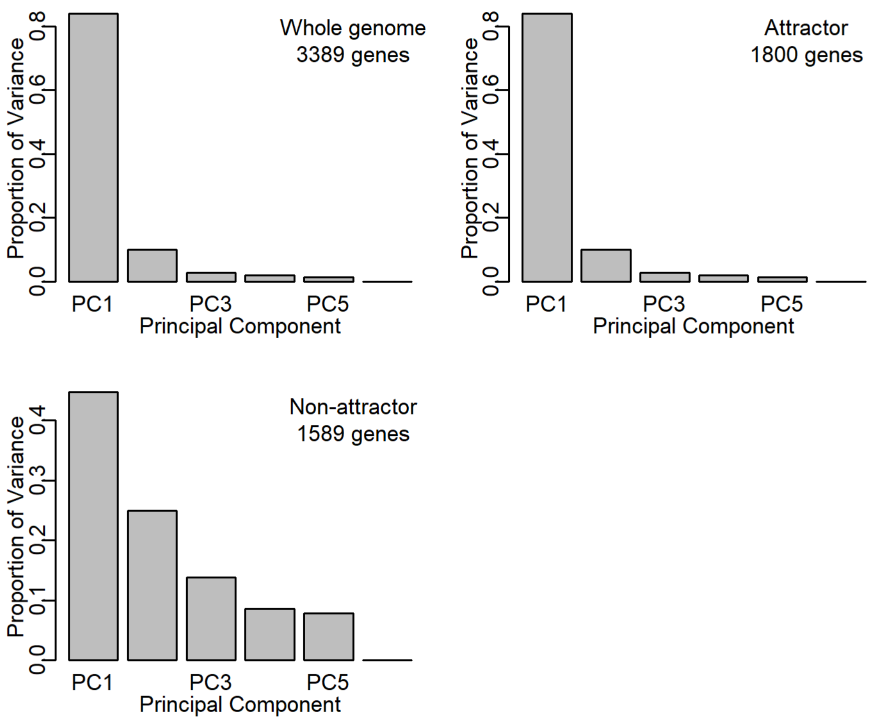

Figure S5B

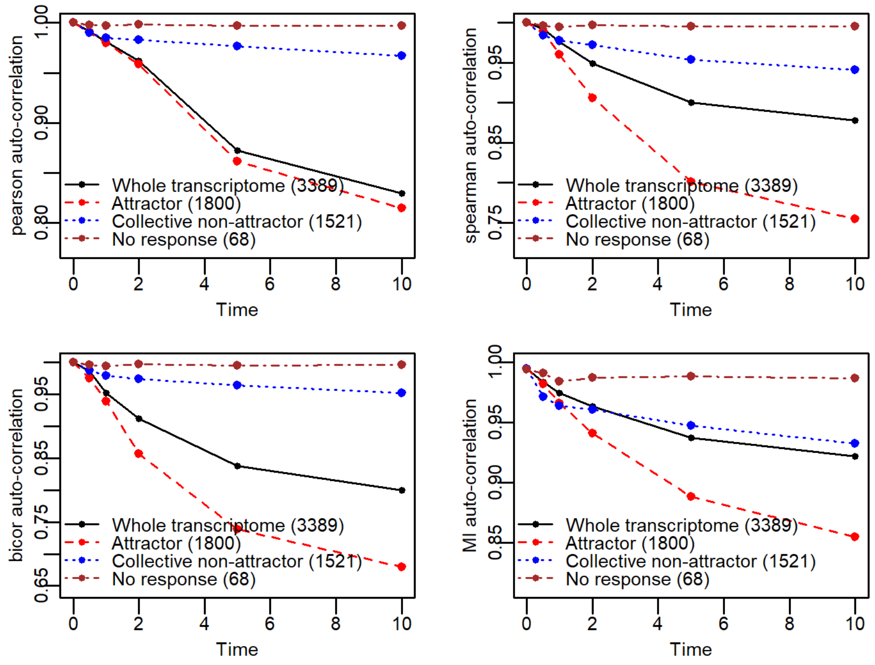

Figure S6A

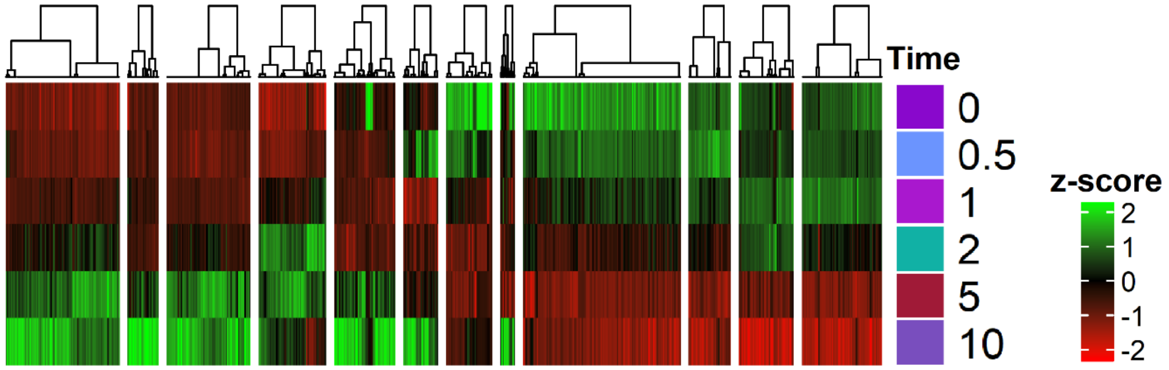

Figure S6B

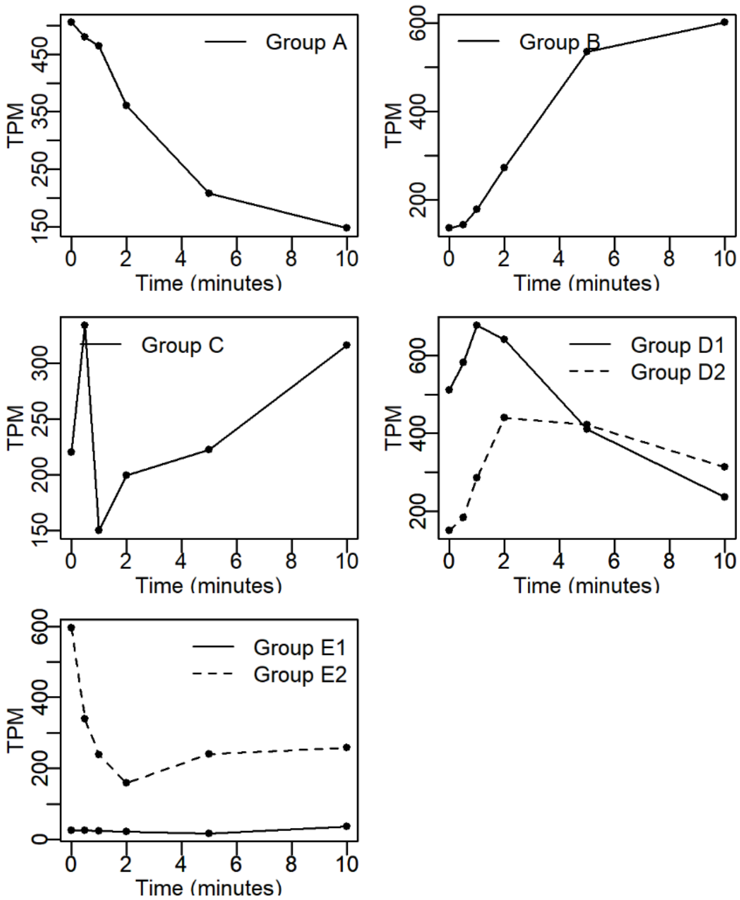

Figure S6C

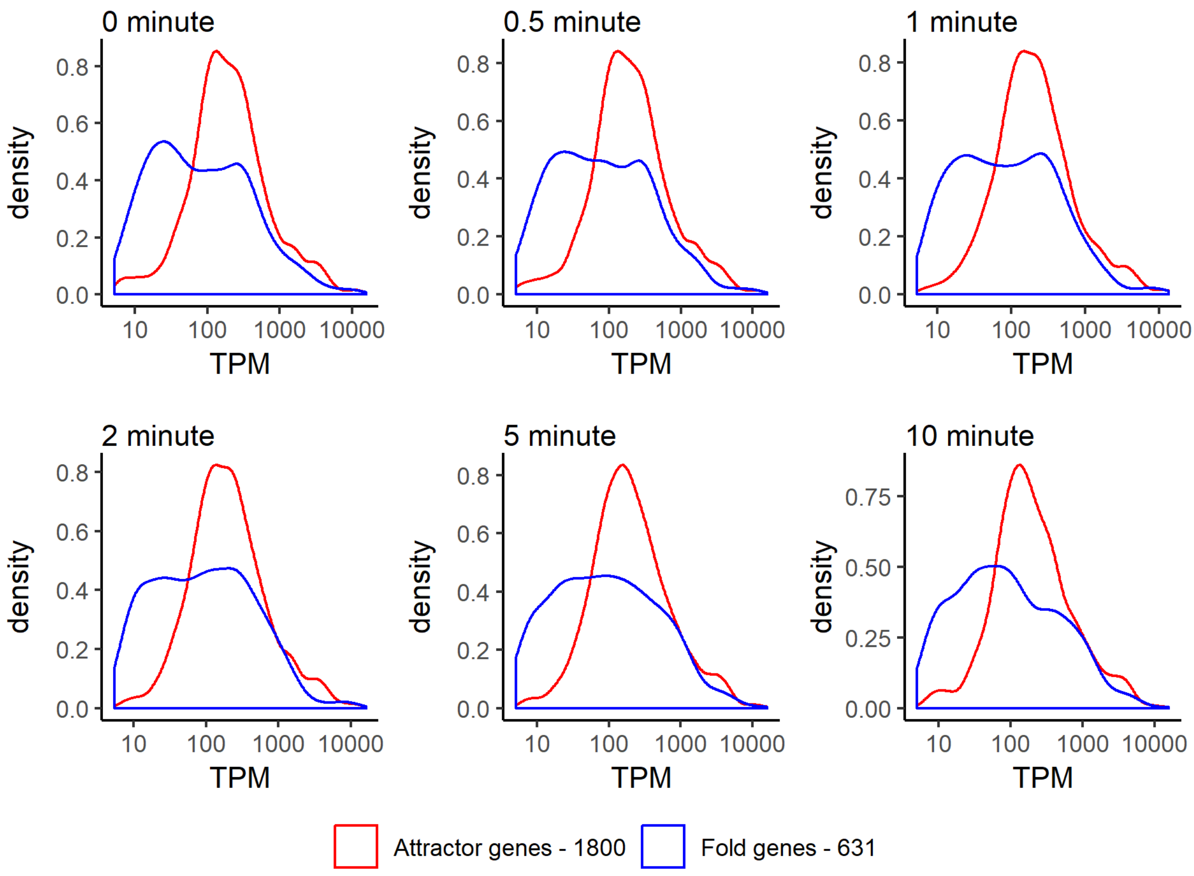
0 minute
0.5 minute
1 minute
2 minute
5 minute
10 minute
density
TPM
0.0
0.2
0.4
0.6
0.8
0.00
0.25
0.50
0.75
10
100
1000
10000
Attractor genes - 1800
Fold genes - 631

Supplemental table legend

***Table S1*. Alkaike Information Criterion (AIC) Values for 3 replicates (denoted as sample a, b, and c) in 6 time points (0, 0.5, 1, 2, 5, 10 minutes).**

***Table S2*. Biological processes enriched in the 6 major expression patterns of attractor genes with their associated genes.** (Table S2 is provided in the supplemental Excel file)

***Table S3.* Gene ID and their coding protein name for each temporal expression group of attractor genes.** (A) Group A, with 72 uncharacterized genes. (B) Group B, with 61 uncharacterized genes. (C) Group C, with 77 uncharacterized genes. (D) Group D, with 19 uncharacterized genes. (E) Group E, with 33 uncharacterized genes. (F) Group F, with 73 uncharacterized genes. Genes highlighted in yellow have novel or uncharacterized functions. Note: the tRNA and rRNA genes in these groups are marked as unmapped genes in Entrez Gene identifier database during Gene Ontology enrichment analysis. (Table S3 is provided in the supplemental Excel file)

***Table S4.* Novel gene ID and their coding protein name for each temporal expression group of attractor genes.** (A) Group A, with 72 uncharacterized genes. (B) Group B, with 61 uncharacterized genes. (C) Group C, with 77 uncharacterized genes. (D) Group D, with 19 uncharacterized genes. (E) Group E, with 33 uncharacterized genes. (F) Group F, with 73 uncharacterized genes. (Table S4 is provided in the supplemental Excel file)

***Table S5*: List of 94 refined *attractor* genes for all groups.** Refined attractor genes have expression over 500TPM and at least 3-fold change between any 2 time points. (Table S5 is provided in the supplemental Excel file)

***Table S6*: Gene ID and their coding protein name for *non-attractor* genes with two-fold expression change genes.** Novel or uncharacterized genes are highlighted in yellow. (Table S6 is provided in the supplemental Excel file)

Table S1

Data column	Lognormal	Pareto	Burr	Loglogistic	Weibull	Gamma	min AIC
Sample a - 0 minute	49098.75	49255.19	49215.14	49247.69	50180.74	51374.37	Lognormal
Sample a - 0.5 minute	48344.28	48551.5	48507.93	48516.81	49039.09	50156.55	Lognormal
Sample a - 1 minute	48214.76	48424.09	48365.63	48381.71	48876.56	50003.22	Lognormal
Sample a - 2 minute	48364.88	48566.01	48529.36	48534.96	49078.37	50197.62	Lognormal
Sample a - 5 minute	48220.25	48449.67	48387.86	48400.01	48866.07	49905.46	Lognormal
Sample a - 10 minute	47809.02	48025.41	47964.63	47973.92	48494.05	49620.65	Lognormal
Sample b - 0 minute	48642.39	48836.06	48789.4	48803.99	49292.29	50337.54	Lognormal
Sample b - 0.5 minute	48491.43	48704.59	48633.85	48657.3	49097.76	50127.62	Lognormal
Sample b - 1 minute	48841.43	49051.21	49033.92	49034.86	49603.09	50638.59	Lognormal
Sample b - 2 minute	48526.78	48750.51	48671.86	48693.55	49125.86	50163.96	Lognormal
Sample b - 5 minute	48473.24	48706.49	48642.12	48656.2	49117.31	50172.92	Lognormal
Sample b - 10 minute	49269.51	49441.38	49439.1	49443.25	50215.4	51289.65	Lognormal
Sample c - 0 minute	48257.48	48423.97	48409.13	48412.21	49017.74	50139.47	Lognormal
Sample c - 0.5 minute	48983.88	49168.25	49156.31	49157.84	49753.5	50846.05	Lognormal
Sample c - 1 minute	48741.22	48971.46	48909.27	48926.51	49384.77	50424.94	Lognormal
Sample c - 2 minute	48488.08	48693.12	48650.92	48661.23	49186.1	50327.67	Lognormal
Sample c - 5 minute	48799.45	48976.71	48976.61	48974.65	49636.51	50734.56	Lognormal
Sample c - 10 minute	48829.55	49006.02	49002	49000.89	49646.14	50797.74	Lognormal
